# Beta2-adrenoreceptor agonist clenbuterol produces transient decreases in alpha-synuclein mRNA but no long-term reduction in protein

**DOI:** 10.1038/s41531-022-00322-x

**Published:** 2022-05-24

**Authors:** Joseph R. Patterson, Warren D. Hirst, Jacob W. Howe, Christopher P. Russell, Allyson Cole-Strauss, Christopher J. Kemp, Megan F. Duffy, Jared Lamp, Andrew Umstead, Michael Kubik, Anna C. Stoll, Irving E. Vega, Kathy Steece-Collier, Yi Chen, Anne C. Campbell, Catherine L. Nezich, Kelly E. Glajch, Caryl E. Sortwell

**Affiliations:** 1 Department of Translational Neuroscience, Michigan State University, Grand Rapids, MI USA; 2 Neurodegenerative Diseases Research Unit, Biogen, Cambridge, MA USA; 3 Neuroscience Program, Michigan State University, East Lansing, MI USA; 4 Cell and Molecular Biology Department, Grand Valley State University, Allendale, MI USA; 5 Department of Pharmacology and Toxicology, Michigan State University, East Lansing, MI USA; 6 Mercy Health Hauenstein Neuroscience Medical Center, Grand Rapids, MI USA

**Keywords:** Parkinson's disease, Target validation

## Abstract

β2-adrenoreceptor (β2AR) agonists have been associated with a decreased risk of developing Parkinson’s disease (PD) and are hypothesized to decrease expression of both alpha-synuclein mRNA (*Snca)* and protein (α-syn). Effects of β2AR agonist clenbuterol on the levels of *Snca* mRNA and α-syn protein were evaluated in vivo (rats and mice) and in rat primary cortical neurons by two independent laboratories. A modest decrease in *Snca* mRNA in the substantia nigra was observed after a single acute dose of clenbuterol in rats, however, this decrease was not maintained after multiple doses. In contrast, α-syn protein levels remained unchanged in both single and multiple dosing paradigms. Furthermore, clenbuterol did not decrease *Snca* in cultured rat primary cortical neurons, or decrease *Snca* or α-syn in mice. Additionally, compared to the single-dose paradigm, repeat dosing resulted in substantially lower levels of clenbuterol in plasma and brain tissue in rodents. Based on our observations of a transient decrease in *Snca* and no effect on α-syn protein in this preclinical study, these data support the conclusion that clenbuterol is not likely a viable disease-modifying strategy for PD.

## Introduction

The formation and accumulation of intracellular Lewy Bodies (LBs) is a key pathological feature of Parkinson’s disease (PD). A principal component of LBs is the protein alpha-synuclein (α-syn), specifically α-syn, which is misfolded, aggregated and phosphorylated at serine 129 (pSyn)^1–5^. Aggregation of α-syn occurs in idiopathic and most familial forms of PD. Mutations in the gene that encodes α-syn (hereafter referred to as *SNCA* when discussing humans and *Snca* when discussing rodents), as well as *SNCA* duplication and triplication, can increase the propensity of α-syn to aggregate^6–9^. *SNCA* duplication or triplication produces a gene dosage effect, with the triplication compared to duplication leading to earlier symptom onset and more rapid disease progression^10^.

Although α-syn is commonly discussed as a pathological cause that forms aggregates associated with disease state, the monomeric soluble form is a crucial component of neuronal function. α-syn has been proposed to have roles in synaptic vesicle trafficking and neurotransmission, mitochondrial function, dopamine biosynthesis and processing, chaperone protein function, DNA repair, and gene expression, among other predicted functions^11–20^. Inadequate levels of α-syn have the potential to be detrimental to neurons and neuronal function, which is why a complete knockdown or elimination of α-syn would likely induce undesirable side-effects^21,22^. Indeed, transgenic mouse models have shown that knockout of *Snca* alone are insufficient to produce effects^23,24^, and simultaneous knockouts of *Snca*, *Sncb*, and *Sncg* are required to produce robust deficits^25–27^. These transgenic models come with the caveat of significant compensatory mechanisms associated with the lack of *Snca* from birth, with as many as 369 differentially expressed genes reported in *Snca-/-* mice^28^.

Cumulative data suggests that moderate reductions in α-syn hold the best potential as a therapeutic strategy, i.e., decreasing α-syn levels while maintaining the critical threshold required to avoid potential loss-of-function effects in neurons. Nevertheless, modulation of α-syn levels in the brain is not trivial. Animal models in which gene expression was modified via shRNA or antisense nucleotides have had mixed results, with some studies showing toxicity^29,30^, while others do not^31–34^. As an alternative, pharmaceutical interventions may provide a superior approach to produce a modest but physiologically meaningful decrease in α-syn.

Drugs targeting the β2-adrenoreceptor (β2AR), have been reported to modulate α-syn levels and affect PD risk^35–40^. Population-based studies have reported an association between β2AR agonist use and decreased PD risk^37,38^, although others have challenged these results, finding β2AR agonists alone do not impact PD risk^39,40^. Beyond the inconsistent results surrounding these association studies, preclinical evidence in rodents and cell culture suggest that drugs acting on β2AR can influence α-syn expression^37^. Specifically, β2AR agonists are reported to decrease *Snca* mRNA and α-syn protein in vivo and in vitro^37^. Conversely, β2AR antagonists have been reported to increase *Snca* mRNA and α-syn protein in culture^37^. Alteration of α-syn levels via β2AR is hypothesized to occur through transcriptional regulation by acetylation or deacetylation of H3K27 along the *Snca* promoter and enhancer sites; with β2AR agonists or β2AR antagonists leading to a decrease or increase in the permissive acetylated H3K27, respectively, thus impacting transcription of *Snca*^37^. Further, the β2AR agonist clenbuterol has been reported to be beneficial in models that recapitulate features of PD. Specifically, clenbuterol decreases *SNCA* mRNA and α-syn protein in *SNCA*-triplication human iPSCs, and prevents neurodegeneration in the 1-methyl-4-phenyl-1,2,3,6-tetrahydropyridine (MPTP) mouse model of PD^37^.

The present set of experiments is the culmination of work performed independently in laboratories at Michigan State University (MSU) and Biogen. In these experiments, we sought to replicate and build on the original findings that β2AR agonists, specifically clenbuterol, reduce *Snca* mRNA and α-syn protein in rodents and cell culture^37^. However, the clenbuterol dosing paradigm used in rats did not result in either decreased α-syn protein in acute studies or changes in either *Snca* or α-syn protein after multiple doses. Direct replication studies in mice mirrored results observed in rats, with no significant reduction in *Snca* or α-syn protein observed following single or multiple doses of clenbuterol. Additionally, there was no change in *Snca* in rat primary cortical cultures. Taken together, the results suggest that any reductions of α-syn transcript following clenbuterol are transient and do not result in reduced abundance of α-syn protein. Thus, we conclude that clenbuterol is unlikely to translate to an effective disease-modifying PD treatment.

## Results

### Clenbuterol administration in rats

**Fig. 1:**
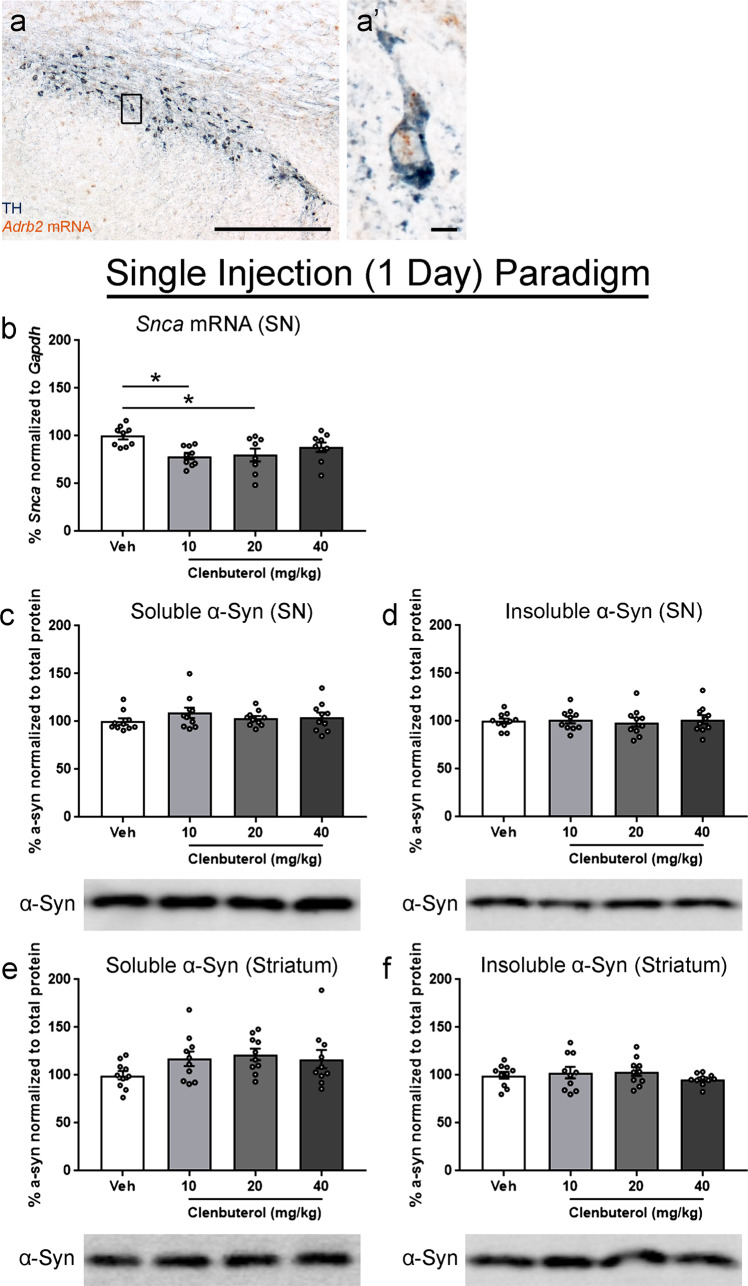
Effects of acute clenbuterol on *Snca* transcript and α-syn protein in rats. Rats received a single intraperitoneal injection of 10, 20, or 40 mg/kg clenbuterol (clen) or saline vehicle (veh) and tissue was collected 24 h post-injection. **a** Representative image of in situ hybridization for *Adrb2* mRNA, which encodes β2AR, colocalized within a tyrosine hydroxylase (TH) immunoreactive neurons in the SNpc of an untreated rat. **b** *Snca* normalized to *Gapdh* and measured via ddPCR. **c** Readily soluble α-syn fraction isolated from the SN. **d** Initially insoluble α-syn fraction isolated from the SN, solubilized with a stronger lysis buffer. **e** Readily soluble α-syn fraction isolated from the striatum. **f** Initially insoluble α-syn fraction isolated from the striatum, solubilized with a stronger lysis buffer. All protein fractions were measured via western blot, graphed as percent of control, and representative blots are shown below the respective graph. Columns indicate the group means, circles represent individual data points (*n* = 10 per group before outlier removal), error bars represent ±1 standard error of the mean. An asterisk represents significance (*p* ≤ 0.05). Outliers were removed based on the absolute deviation from the median method. In **b**, one sample was removed the veh, 10, and 40 mg/kg groups, and two samples were removed from the 20 mg/kg group. No other outliers were removed.

We first confirmed that rat nigrostriatal dopamine neurons express β2AR using in situ hybridization (*Adrb2*) combined with immunohistochemistry (IHC) for tyrosine hydroxylase (TH) in the substantia nigra (SN). Abundant *Adrb2* mRNA puncta were observed within individual TH immunoreactive (THir) nigral neurons (Fig. 1a).

Based on intraperitoneal (i.p.) injections yielding significantly higher levels of clenbuterol in the CSF compared to subcutaneous injections (Supplementary Fig. 1), i.p. administration was selected. To mirror previous studies^37^, we administered a single injection (i.p.) of 10, 20, or 40 mg/kg clenbuterol or vehicle (saline), *Snca* mRNA and α-syn protein were assessed in the SN, and α-syn protein in the striatum 24 h later (see experimental design, Supplementary Fig. 2). A modest, yet significant decrease of *Snca* in the SN was observed using droplet digital PCR (ddPCR) in rats that received a single injection of 10 or 20 mg/kg clenbuterol (Fig. 1b). *Snca* was reduced ~20% by both the 10 mg/kg and 20 mg/kg dose, whereas no significant reduction in *Snca* was observed in rats receiving the highest dose, 40 mg/kg (Fig. 1b).

Preparation of rat nigral and striatal samples for measurement of α-syn protein was performed using a two-step lysis. The first step used a weak lysis buffer and pestle homogenization, to replicate protein isolation procedures as previously reported^37^, referred to as “soluble α-syn”. The second step used a strong radio-immunoprecipitation assay (RIPA) buffer and sonication of the pellet remaining from the first step, referred to as “insoluble α-syn”. We did not observe any impact of single i.p. clenbuterol injections to rats on either the soluble or insoluble α-syn protein levels in the SN using any dose of clenbuterol (Fig. 1c, d). Similarly, western blots for the readily soluble and insoluble α-syn in the striatum showed no change with clenbuterol administration (Fig. 1e, f).

**Fig. 2:**
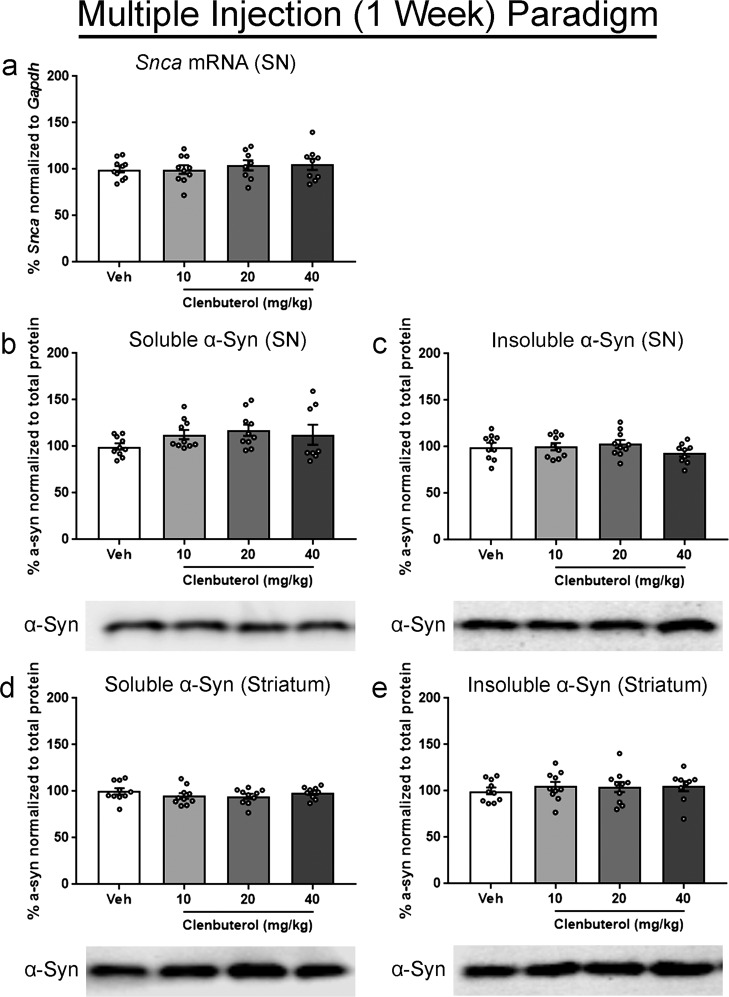
Assessment of week-long clenbuterol treatment on *Snca* transcript and α-syn protein in rats. Rats received intraperitoneal injections of 10, 20, or 40 mg/kg clenbuterol (clen) or saline vehicle (veh) every 48 h. Tissue was collected on the 7th day, 24 h after the final injection. Prior to each injection, rats were weighed to calculate the volume of drug or vehicle required for the dose. **a** *Snca* normalized to *Gapdh* and measured via ddPCR. **b** Readily soluble α-syn fraction isolated from the SN. **c** Initially insoluble α-syn fraction isolated from the SN, solubilized with a stronger lysis buffer. **d** Readily soluble α-syn fraction isolated from the striatum. **e** Initially insoluble α-syn fraction isolated from the striatum, solubilized with a stronger lysis buffer. All protein fractions were measured via western blot, graphed as percent of control, and representative blots are shown below the respective graph. Columns indicate the group means, circles represent individual data points (*n* = 10 per group before outlier removal), error bars represent ±1 standard error of the mean. An asterisk represents significance (*p* ≤ 0.05). Outliers were removed based on the absolute deviation from the median method. In **a**, two samples were removed from the 20 mg/kg, and one sample removed from the 40 mg/kg groups. In **b**, two samples were removed from the 40 mg/kg group. In **c** and **e**, one sample was removed from the 40 mg/kg group. No other outliers were removed.

The lack of decrease in α-syn protein observed after single clenbuterol administration, in contrast to the observed decrease in transcript, could be due to inadequate protein degradation/clearance over the 24-h interval. This suggests that given adequate time, reductions in α-syn protein may be observable. Thus, to determine the impact of clenbuterol administration over a longer, multiple dosing paradigm, we examined *Snca* mRNA and α-syn protein levels with 1 week of repeated clenbuterol administration (Supplementary Fig. 3). Rats received an i.p. injection every 48 h for a total of four doses, based on the reported 30 h half-life of clenbuterol in plasma with oral administration^41^. Rats in clenbuterol groups demonstrated a noticeable weight loss compared to controls with all three doses at 2, 4, 6, and 7 days into the injection series (Supplementary Fig. 4). When *Snca* mRNA was examined, we observed no change in *Snca* in the SN with any dose in the multiple injection paradigm, in contrast to results from the single injection paradigm (Fig. 2a). Western blots for soluble and insoluble α-syn in the SN, and the striatum also showed no change in response to any clenbuterol dose (Fig. 2b–e).

### Clenbuterol administration in mice

Mittal et al.^37^ have previously examined the effects of clenbuterol on α-syn expression in mice. In their study, mice received either a single i.p. injection of clenbuterol (10 mg/kg) dissolved in saline or an equal volume of saline as a control. Animals were processed 24 h following the injection and a 20–50% decrease in both *Snca* mRNA and α-syn protein was reported in the SN^37^. To address whether clenbuterol effects are specific to mice, we performed a study using parameters identical to what were previously reported (Supplementary Fig. 2)^37^.

**Fig. 3:**
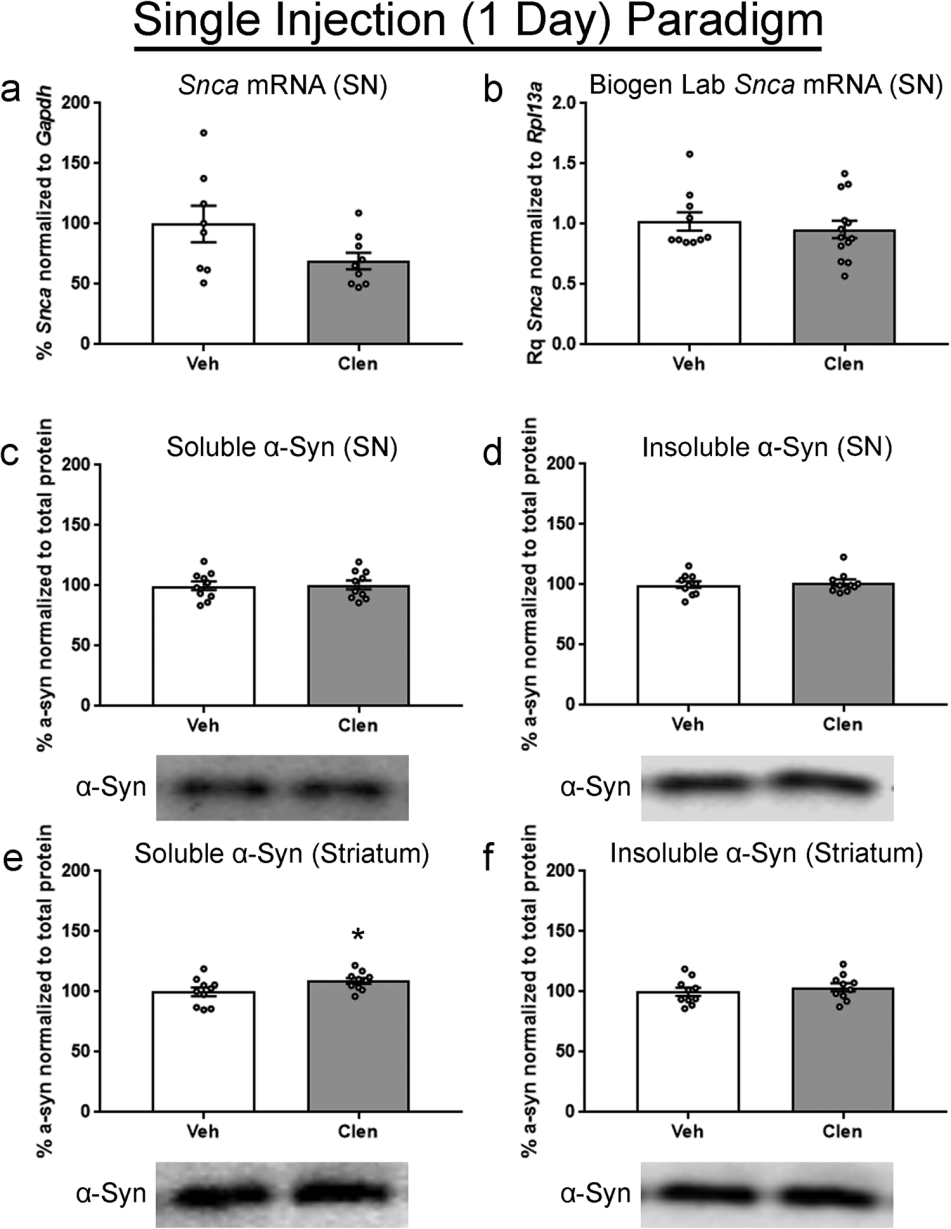
Effects of acute clenbuterol on *Snca* transcript and α-syn protein in mice. Mice received a single intraperitoneal injection of 10 mg/kg clenbuterol (clen) or saline vehicle (veh) and tissue was collected 24 h post-injection. **a** *Snca* normalized to *Gapdh* and measured via ddPCR. **b** Results from the Biogen lab, *Snca* mRNA normalized to *Rpl13a* mRNA and measured via RT-qPCR. **c** Readily soluble α-syn fraction isolated from the SN. **d** Initially insoluble α-syn fraction isolated from the SN, solubilized with a stronger lysis buffer. **e** Readily soluble α-syn fraction isolated from the striatum. **f** Initially insoluble α-syn fraction isolated from the striatum, solubilized with a stronger lysis buffer. All protein fractions were measured via western blot, graphed as percent of control, and representative blots are shown below the respective graph. Columns indicate the group means, circles represent individual data points (*n* = 10 per group before outlier removal), error bars represent ±1 standard error of the mean. An asterisk represents significance (*p* ≤ 0.05). Outliers were removed based on the absolute deviation from the median method. In **a**, two samples were removed from the veh, and one sample removed from the clen groups. No other outliers were removed.

In the SN, *Snca* mRNA in clenbuterol treated animals trended towards a decrease, but the effect was not significant (Fig. 3a). Results from a replicate study performed at Biogen using the same single dose paradigm and timepoint also observed no change in *Snca* mRNA (Fig. 3b). Assessment of α-syn protein by western blot was again performed using a two-step lysis. In both soluble and insoluble fractions, we observed no decrease in α-syn protein in the SN (Fig. 3c, d). In the striatum, there was a significant albeit slight increase in soluble α-syn in the clenbuterol group, which was 109.1% ± 2.41 of control (Fig. 3e). There was, however, no detectable increase of insoluble α-syn in the clenbuterol treated group (Fig. 3f).

**Fig. 4:**
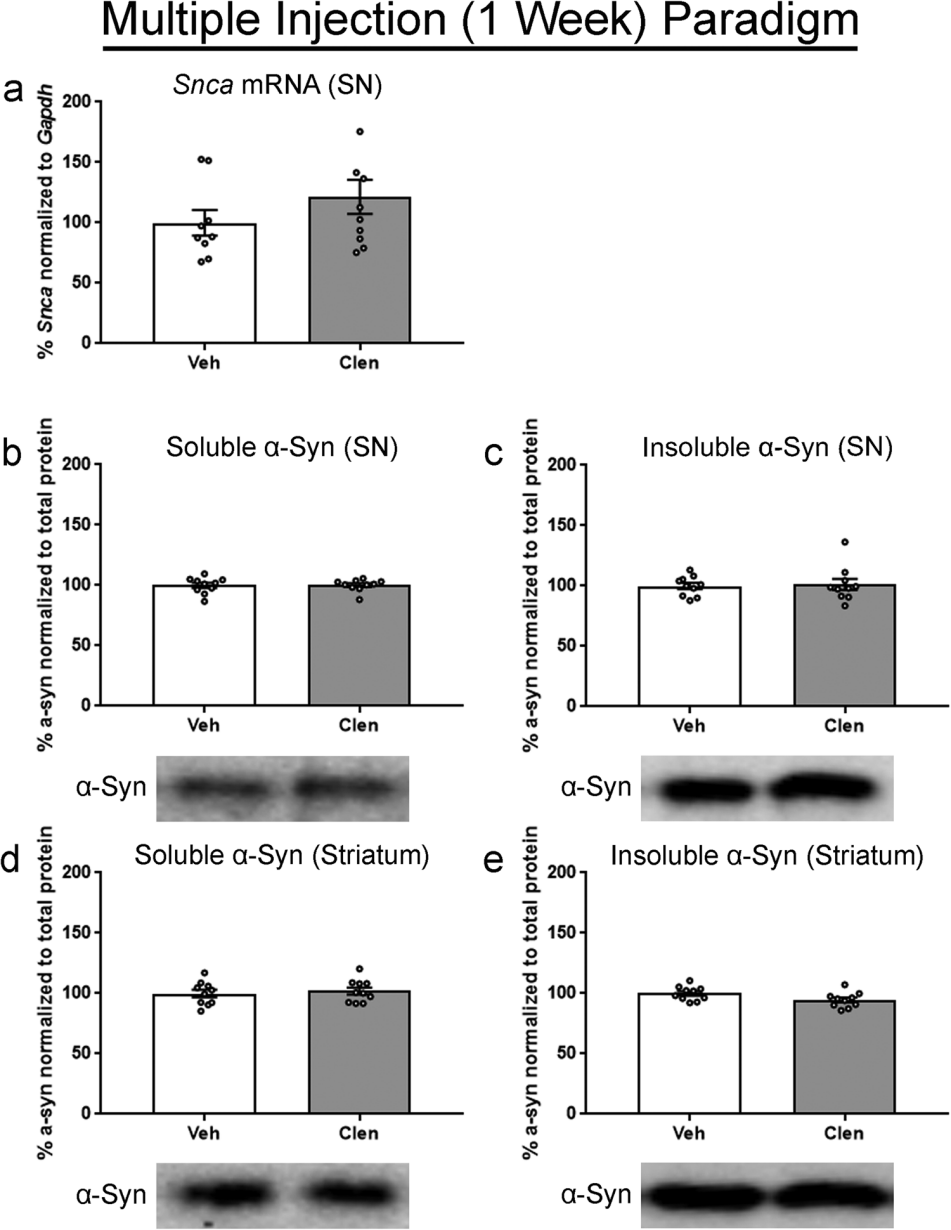
Assessment of week-long clenbuterol treatment on *Snca* transcript and α-syn protein in mice. Mice received intraperitoneal injections of 10 mg/kg clenbuterol (clen) or saline vehicle (veh) every 48 h. Tissue was collected on the 7th day, 24 h after the final injection. **a** *Snca* normalized to *Gapdh*, and measured via ddPCR. **b** Readily soluble α-syn fraction isolated from the SN. **c** Initially insoluble α-syn fraction isolated from the SN, solubilized with a stronger lysis buffer. **d** Readily soluble α-syn fraction isolated from the striatum. **e** Initially insoluble α-syn fraction isolated from the striatum, solubilized with a stronger lysis buffer. All protein fractions were measured via western blot, graphed as percent of control, and representative blots are shown below the respective graph. Columns indicate the group means, circles represent individual data points (*n* = 10 per group before outlier removal), error bars represent ±1 standard error of the mean. An asterisk represents significance (*p* ≤ 0.05). Outliers were removed based on the absolute deviation from the median method. In **a**, one sample was removed from the veh and clen groups. No other outliers were removed.

Allowing for the possibility that onset of clenbuterol effects could be delayed, we examined the impact of multiple injections of 10 mg/kg clenbuterol in mice over 1 week (Supplementary Fig. 3). There was no change in *Snca* mRNA in the SN after 1 week treatment paradigm (Fig. 4a). Similarly, we observed no impact of multiple injections on α-syn protein in the mouse SN by western blot, in either the soluble or insoluble fraction (Fig. 4b, c). Similarly, there was no change in α-syn insoluble or insoluble fractions in the striatum after 1 week (Fig. 4d, e). Compared to vehicle, mice receiving clenbuterol lost weight early in this dosing paradigm but recovered and did not show significant weight loss at and beyond day four of injections (Supplementary Fig. 4).

**Fig. 5:**
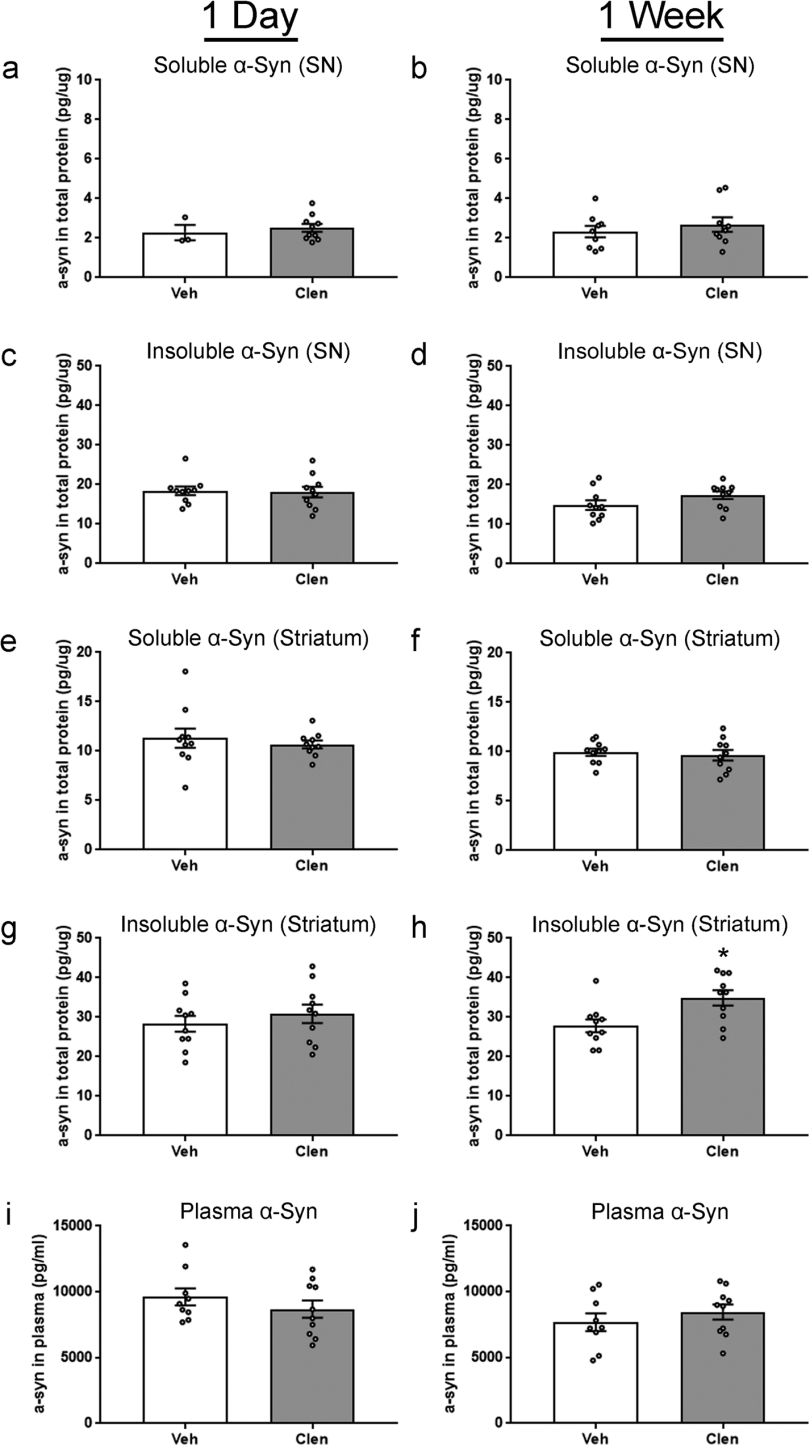
Assessment of mouse α-syn protein from SN, striatum, and plasma by using ELISA. A commercially available ELISA kit was used to measure α-syn in the plasma and the remaining protein lysates from the 1 day and 1 week mouse 10 mg/kg dosing paradigms. Readily soluble α-syn isolated from the SN at **a** 1 day, and **b** 1 week. Initially insoluble α-syn isolated from SN, solubilized with a stronger lysis buffer at **c** 1 day, and **d** 1 week. Soluble α-syn from the striatum at **e** 1 day and **f** 1 week. Insoluble α-syn from the striatum at **g** 1 day and **h** 1 week. Total α-syn in plasma at **i** 1 day and **j** 1 week. Columns indicate the group means, circles represent individual data points (*n* = 10 per group before outlier removal), error bars represent ±1 standard error of the mean. An asterisk represents significance (*p* ≤ 0.05). Outliers were removed based on the absolute deviation from the median method. In **a**, there were no outliers removed, the small sample size in the veh group was due to the limited amount of protein remaining for the assay. In **b**, one sample was removed from the veh and clen groups. In **i**, **j** one sample was removed from the veh group. No other outliers were removed.

As decreases in α-syn protein were shown via an enzyme-linked immunoassay (ELISA) in the original report^37^, we also analyzed mouse cohorts using a commercially available ELISA. We observed no impact of clenbuterol on soluble or insoluble α-syn protein levels in either the SN or striatum at 24 h in the acutely treated mice (Fig. 5a, c, e, g). In the 1-week paradigm, there was no change in the soluble or insoluble α-syn protein levels in the SN, and no change in soluble α-syn protein levels in the striatum (Fig. 5b, d, f). We did observe a significant increase in α-syn protein in the striatum in the insoluble fraction, 34.9 ± 1.9 pg/µg in the 1-week clenbuterol group compared to 27.9 ± 1.6 pg/µg in the vehicle control group (Fig. 5h). The ELISAs and a portion of the western blots in the original report used a different α-syn antibody (Millipore, MABN1817, α-syn clone 2F12) than we used (Abcam, ab212184). Unfortunately, we did not have lysate remaining from the SN to do a second assessment of α-syn levels using this other antibody. However, to address this difference, we further interrogated the soluble fraction from the striatum. Again, there was no change by western blot in the stratum soluble fraction in the 24 h or 1-week paradigm using the MABN1817 antibody (Supplementary Fig. 5).

One remaining difference between our sample preparation and that of Mittal et al.^37^ was that all rats and mice in our studies were perfused with saline to eliminate the potential of α-syn protein in blood to which would contribute to brain tissue α-syn levels. To determine whether clenbuterol impacts blood α-syn protein levels, we measured α-syn protein in plasma. We observed no differences in plasma α-syn protein levels in mice for either the 24 h or 1-week paradigm following 10 mg/kg clenbuterol injection (Fig. 5i, j).

Collectively, we observed, in both rats and mice, that the ability of clenbuterol to reduce α-syn was limited to a reduction in α-syn transcript after a single injection, which was no longer observable in the multiple injection paradigm. β2AR is subject to desensitization with chronic agonist exposure^42^. Desensitization of β2AR can be followed by the internalization and/or downregulation of the receptor. To examine potential changes in β2AR, tissue from the striatum and hippocampus were first homogenized in a strong lysis buffer and assessed by western blot. Total β2AR protein was unchanged after a week of clenbuterol treatment in the striatum and the hippocampus (Supplementary Fig. 6a, b). A marker for internalized β2AR is the phosphorylation of serines 355/356 by G protein-coupled receptor kinase^43^. Phosphorylated-β2AR (ser355/356) was unchanged after a week of clenbuterol treatment in the striatum, as well as in the hippocampus (Supplementary Fig. 6c, d). Taken together, these results do not support that internalization or downregulation of the β2AR receptor occurred with repeat clenbuterol administration. However, these findings cannot rule out functional desensitization, where agonist binding produces a weaker response.

An additional endpoint we examined was protein levels of the dopamine transporter (DAT), which is required for the uptake of MPP^+^, the toxic metabolite of MPTP^44,45^. Not previously measured in the context of clenbuterol use, a clenbuterol-mediated decrease in DAT could potentially explain the neuroprotective effects reported for clenbuterol in the MPTP mouse model^37^. In the striatum of mice, a single 10 mg/kg injection of clenbuterol significantly reduced DAT protein levels by ~23% compared to control (Supplementary Fig. 7a). This reduction in striatal DAT was not observed in the multiple injection paradigm (Supplementary Fig. 7b). Additionally, striatal DAT protein was unchanged in rats after a single clenbuterol injection, or after multiple injections (Supplementary Fig. 7c, d).

### Clenbuterol levels in mice and rats

**Fig. 6:**
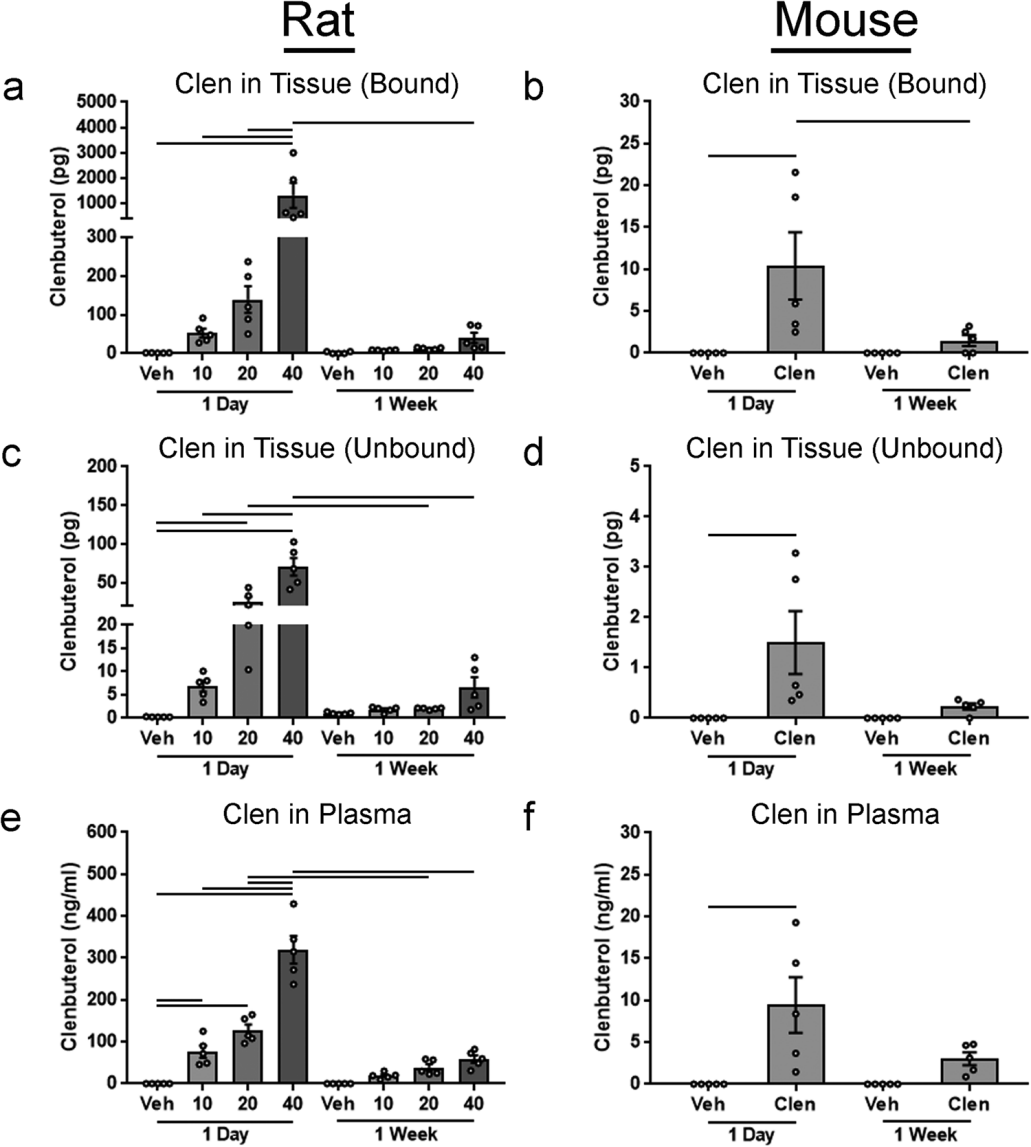
Measurement of clenbuterol levels in tissue and plasma of mice and rats. Tissue from the hippocampus, and plasma from mice and rats in the 1 day and 1-week paradigms were collected. Tissue was treated to separate the clenbuterol (clen) that was and was not interacting with proteins, producing a bound and unbound clenbuterol fraction respectively. Bound clenbuterol in **a** rats and **b** mice. Unbound clenbuterol in the **c** rat and **d** mice. Total clenbuterol in the plasma from **e** rats and **f** mice. Columns indicate the group means, circles represent individual data points (*n* = 5 per group), error bars represent ±1 standard error of the mean. A line connecting columns indicates significance between groups (*p* ≤ 0.05).

Clenbuterol in the plasma and brain (hippocampus) was measured to determine levels present in both rats and mice. In both the single and multiple injection paradigms in rats, a dose-dependent increase in bound and unbound clenbuterol levels was observed in plasma and tissue, (Fig. 6a, c, e). Strikingly, clenbuterol levels measured in animals receiving multiple injections, were significantly lower than their single injection counterparts (Fig. 6a, c, e). Clenbuterol levels in the bound fraction of the single injection groups were ~7, 14, and 32 times higher than their respective 10, 20, and 40 mg/kg multiple injection counterparts. Clenbuterol levels in the unbound fraction of the single injection groups was ~4, 10, and 10 times higher than their respective 10, 20, and 40 mg/kg multiple injection counterparts. Total clenbuterol levels in plasma of the single injection groups was ~4, 3, and 10 times higher than their respective 10, 20, and 40 mg/kg multiple injection counterparts. While the dose-dependent increase in clenbuterol levels in plasma and tissue was expected, the decreased levels after a series of injections compared to a single injection were not. Similarly in mice, clenbuterol in plasma and tissue was lower after multiple injections compared to a single injection (Fig. 6b, d, f). In the single injection group, clenbuterol levels in the bound and unbound fractions were ~7, and 3 times higher in plasma, respectively, compared to the multiple injection group. Given that both single and multiple injection groups were sacrificed ~24 h after their final injection, the expectation would be that clenbuterol levels would be either comparable or higher in the multiple injection group due to accumulation.

### Clenbuterol administration in primary rat cortical neurons

**Fig. 7:**
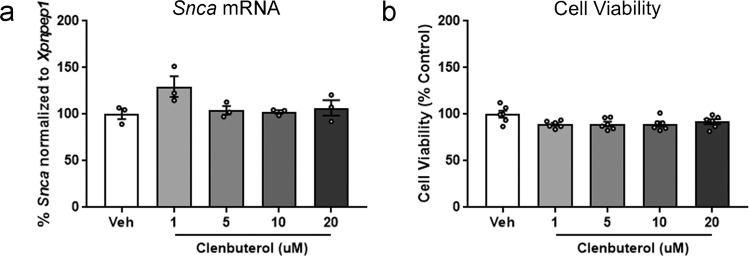
Assessment of the effects of clenbuterol on *Snca* transcript in primary rat cortical neurons. E18 primary rat cortical neurons were treated with clenbuterol or vehicle. **a** *Snca* measured via RT-qPCR in neurons exposed at DIV11 and harvested 2 days later. **b** Cell viability of primary neurons exposed at DIV11 and examined 2 days later. Columns indicate the group means, circles represent individual data points from the same experiment, error bars represent ±1 standard error of the mean. An asterisk represents significance (*p* ≤ 0.05).

Similar to Mittal et al.^37^, primary rat cortical neurons from E18 Sprague-Dawley rat embryos were treated for 2 days with clenbuterol (1, 5, 10, or 20 µM), then assessed for *Snca* mRNA by RT-qPCR. In contrast to the previous report^37^, clenbuterol had no effect on α-syn transcript at any concentration (Fig. 7a). The corresponding cell viability assay showed no significant toxicity associated with any of the concentrations of clenbuterol used. (Fig. 7b).

## Discussion

β2AR agonist and antagonist use with regards to PD and PD risk have been a topic of debate. β2AR agonists were initially shown to decrease PD risk in a Norwegian population^37^. When examined in a USA cohort and adjusted for smoking, β2AR agonist use had no effect on PD risk^39^. A separate cohort from Israel also has reported β2AR agonist use to decrease PD risk, even when smoking, alcohol consumption, residence, and other variables were factored into the analysis^38^. Most recently, β2AR agonists were reported to decrease PD risk in non-diabetic patients while increasing risk in those with diabetes^40^. With regards to β2AR antagonists and PD, there are reports that suggest a link to increased risk^37,38^, those that show no association^46^, and those that observed an association until adjusting for sustained exposure, a 5-year lag between exposure and PD diagnosis, or a specific antagonist propranolol^35,36,39,40^. Propranolol use associated with increased PD risk comes with the caveat that it is used to treat essential tremor, which is itself associated with increased PD risk^38–40,47^. In summation, with no clear consensus in population association data, controlled laboratory studies are critical to provide key insights to determine the potential of β2AR agonists for PD treatment.

In preclinical studies, clenbuterol, has been shown in mice and cell culture studies to decrease α-syn expression through histone modifications, resulting in reductions in *Snca* mRNA and α-syn protein after a single administration^37^. In the present study, we were able to partially replicate the observations of Mittal et al.^37^. Specifically, we observed a significant reduction in *Snca* mRNA or a trend towards decrease, however in neither species did we detect a reduction in α-syn protein following a single injection of clenbuterol to either rats or mice. To understand the impact of repeated clenbuterol administration on *Snca* mRNA and α-syn protein not previously examined by Mittal et al.^37^, we also examined the effects in a multiple injection paradigm. Repeated clenbuterol administration, does not decrease transcript or protein in either mice or rats. Furthermore, clenbuterol similarly has no effect on *Snca* mRNA in primary cortical cultures. Thus, whereas our present results replicate the previous finding that a single administration of clenbuterol can reduce *Snca* mRNA, by expanding our analysis to include the effect of 1-week repeated clenbuterol administration we have revealed that any reduction of *Snca* mRNA appears short term. Importantly, the impact on reducing α-syn protein in either clenbuterol administration paradigm was not observed.

β2AR agonists salmeterol and clenbuterol were shown to have neuroprotective properties against MPTP^37,48^. However, the mechanism by which β2AR agonists provided this neuroprotection is unclear and results come with certain caveats. In both reports, the β2AR agonist was given prior to and during injections of MPTP^37,48^ providing the opportunity for direct drug-insult interaction with MPTP, decreased metabolism of MPTP by MAO-B into its toxic metabolite MPP^+^, decreased uptake of MPP^+^ into neurons via DAT, or reduction of neuroinflammation. In our present study, we observed acute clenbuterol administration was associated with a small reduction in DAT in mice. This raises the possibility that clenbuterol-mediated reduction in DAT levels and/or function interfered with MPP^+^ uptake, thereby decreasing initial MPTP toxicity, and producing a pseudo-neuroprotective effect.

Although MPTP produces selective degeneration of the nigrostriatal pathway, it does not produce robust α-syn pathology^49,50^. Lack of pathological α-syn inclusions in this model suggests that clenbuterol-mediated decreases in pathological α-syn is likely not the mechanism of the neuroprotection reported in the MPTP model^37^. It is noted that transgenic α-syn null mice are resistant to acute and chronic MPTP induced toxicity^51^, potentially implicating loss of α-syn in neuroprotection in the MPTP model. However, resistance to the mitochondrial complex I inhibitor rotenone is not observed in α-syn null embryonic midbrain neurons^51^. These somewhat contradictory results raise the possibility that compensatory gene changes associated with loss of α-syn during development^28^ may participate in neuroprotection from MPTP in α-syn null mice and not loss of α-syn per se.

It is reasonable to expect that chronic β2AR agonist administration would be required to be efficacious for PD disease modification. In the present study, significant reduction in *Snca* mRNA in rats was only observed in the single clenbuterol injection paradigm. Further, at the highest dose in rats (40 mg/kg), no change in mRNA was observed, suggesting an inverted U-shaped dose-effect curve with acute clenbuterol. Decreases in *Snca* mRNA were not detected after multiple injections over a week, suggesting tachyphylaxis associated with clenbuterol. The decrease in clenbuterol levels after repeated administration makes it difficult to determine if sustained β2AR agonism could reduce α-syn protein. It is possible that a different β2AR agonist that is not subject to tachyphylaxis could decrease α-syn. As introduced above, prolonged stimulation of β2AR can result in receptor desensitization, internalization, and downregulation. Clenbuterol doses as low as 0.5 mg/kg twice daily for 4–7 days have been shown to cause functional desensitization of cortical β2ARs^52^. Doses as low as 0.3 mg/kg twice daily for 14 days and 0.25 mg/kg once daily for 10 days have led to receptor desensitization in the rat aorta and uterus, respectively^53,54^. Although we were unable to observe a decrease in β2AR phosphorylation or its abundance in the present study, it remains possible that functional β2AR desensitization occurs following repeated clenbuterol administration. This provides a possible explanation as to why the single dose was associated with decreased α-syn mRNA but not the repeated dosing paradigm. Another possibility for the different effects on α-syn mRNA observed with single vs. multiple dosing paradigms is that repeated clenbuterol administration accelerates its own metabolism. Indeed, in both brain and plasma, we observed lower levels of clenbuterol after the multiple injection paradigm as compared to the single injection. Clenbuterol is a substrate of the cytochrome P450 enzymes CYP1a1 and CYP1a2^55^, and its administration to chickens elevates hepatic microsomal cytochrome P450^56^. Taken together, the transient nature of *Snca* mRNA reduction observed with the single dosing paradigm combined with no detectable reductions in α-syn protein in any paradigm induced by the β2AR agonist clenbuterol, raises questions of whether β2AR agonists would provide any long-term benefits as a risk decreasing or disease-modifying therapy for PD.

Another concept to consider is the effective and toxic doses of the β2AR agonist. In mice, 10 mg/kg of clenbuterol was reported to be required to decrease α-syn, and results in ~20 ng/g of clenbuterol in brain tissue by 24 h post-injection^37^. Although clenbuterol is not FDA-approved for use in humans, it is frequently taken as an over-the-counter supplement for weight loss and muscle growth. In humans, the doses taken are on the order of 20–100 micrograms, with adverse effects such as tachycardia, chest pain, palpitations, tremor, and myocardial ischemia reported in some cases at these doses^57–60^. In rodent models, chronic clenbuterol can be damaging to myocardial tissue^61,62^. These potential adverse side-effects associated with relatively low doses may limit the therapeutic potential of clenbuterol. Similar caution would be recommended with the use of other long-acting β2AR agonists if the goal was to obtain brain levels on par with what is reported to be required in mice to decrease α-syn.

Our present study focused on the effect of clenbuterol specifically on α-syn protein, however, we cannot exclude the potential for β2AR agonists to provide neuroprotection and/or symptomatic benefit in PD via other mechanisms. β2AR agonists have been shown in animal models to increase transport via the large neutral amino acid system, increasing transport of L-tyrosine for dopamine synthesis as well as levodopa across the blood brain barrier^63–68^. The commonly used β2AR agonist for asthma, albuterol, was reported to lead to an improvement in some PD motor symptoms when used as an adjunct therapy to levodopa^63,64^. Additionally, β2AR agonists have been shown to have anti-inflammatory properties associated with neuroprotection. Reduction of ionized calcium-binding adapter molecule 1 (IBA1) and phagocytic marker cluster of differentiation molecule 68 (CD68) positive microglia in response to rotenone^69^, reduced inducible nitric oxide synthase (iNOS) and cluster of differentiation molecule 11B (CD11b) mRNA expression in the hippocampus in response to kainic acid^70^, suppression of microglia proliferation^71^, reduced tumor necrosis factor alpha (TNF-α) and interleukin 6 (IL-6) in response to lipopolysaccharides (LPS)^72,73^, reduced TNF-α and nitric oxide from microglia and neuroprotection from LPS have all been reported^48^. β2AR agonists have also been shown to increase neurotrophic factors, such as nerve growth factor and brain-derived neurotrophic factor^70,74,75^. In addition to models of PD, β2AR agonists have been reported to be neuroprotective in preclinical models of ischemic stroke^76^ and excitotoxicity^70^, promote neurogenesis, dendritic branching and decrease cerebral amyloid plaques in the APP/PS1 transgenic mouse Alzheimer model, and produce motor benefits in the SOD1^G93A^ transgenic ALS mouse model^77^.

In conclusion, our present in vivo results in two species and in vitro results, conducted in two separate laboratories, demonstrate that the ability of clenbuterol to reduce α-syn is transient and limited to modest decreases in α-syn mRNA but not protein. Our results demonstrate that these minimal effects are lost with repeated administration, perhaps due to β2AR receptor desensitization and/or increased drug metabolism. Based on these findings, we conclude that β2AR agonist clenbuterol per se does not have potential to lower α-syn as a disease-modifying strategy for PD.

## Methods

### Michigan state university lab methods

#### Animals (rats and mice)

Three-month-old, male Fischer 344 rats (*n* = 90) were purchased from Charles River Laboratories; eight-week-old male C57BL/6 J mice (*n* = 40) were purchased from the Jackson Laboratory. Rats were housed 1–3 per cage and mice were housed 5 per cage in a room on a 12 h light/dark cycle, and food and water were provided *ad libitum*. All animal work was performed in the Michigan State University Grand Rapids Michigan State Research Center vivarium. All procedures were approved and conducted in accordance with the Michigan State University Institute for Animal Care and Use Committee (IACUC) at Michigan State University.

#### Clenbuterol administration

Clenbuterol HCl (Sigma-Aldrich C5423; Lot# BCBQ2163V) powder was stored at 4 °C prior to use. Approximately 30–60 min before injections, clenbuterol was diluted in sterile 0.9% saline to a concentration of 5 mg/mL based on the free base, then vortexed until completely in solution. Animals were weighed, then received an intraperitoneal (*i.p*.) or subcutaneous (*s.c*.) injection of the appropriate amount of solution based on weight to deliver 10, 20, or 40 mg/kg of clenbuterol. The vehicle control groups received 0.9% saline by weight at a volume equal to the highest volume delivered to the species: mice (10 mg/kg) or rats (40 mg/kg). In multiple-dose studies, injections were performed every other day, taking advantage of the ~30 h half-life^41^ to keep levels of clenbuterol present the entire time. All weighing and injections were performed in the morning, with animals euthanized and tissue/plasma collected the day after the final injection.

#### Sample collection (tissue, CSF, plasma)

Animals were euthanized with a pentobarbital (Beuthanasia-D Special, Merck Animal Health) overdose (30 mg/kg). To collect CSF, rats were secured in a stereotactic instrument modified to hold a butterfly scalp vein set (Exel International, 26708). The needle was lowered into the cisterna magna, CSF extracted, and stored for a short period on ice. CSF was centrifuged (10,000 × g for 10 min at 4 °C) to remove trace amounts of blood, then stored at −80 °C. To collect plasma, cardiac blood was extracted with a heparinized syringe, transferred to a BD Vacutainer™ blood collection tube (BD 367862), inverted to mix, and kept on ice until samples could be processed. Blood was centrifuged (3,000 RPM for 5 min at 4 °C), plasma was collected and stored at −80 °C. Immediately following the collection of cardiac blood, rats were intracardially and mice were transcardially perfused with ice cold heparinized saline (0.9%) to remove blood from the brain that could complicate results. Brains were removed and flash frozen in 2-methylbutane on dry ice, then stored at −80 °C. Brains were mounted and sectioned to the regions of interest on a cryostat at −15 °C, then regions of interest (striatum, substantia nigra, hippocampus) were microdissected and collected in microcentrifuge tubes. Tissue collected for western blots, ELISAs, and mass spectrometry were kept on dry ice then stored at −80 °C. Tissue collected for droplet digital PCR (ddPCR) was added to DNase/RNase free microcentrifuge tubes containing 100 µL TRIzol reagent (Invitrogen 26696026), homogenized with a disposable pestle, volume of TRIzol reagent was brought to 1 mL, mixed by pipetting, frozen on dry ice, and stored at −80 °C.

#### RNA isolation

Samples in TRIzol were thawed on ice and briefly centrifuged to collect all liquid at the bottom of the tube. Phasemaker microcentrifuge tubes (Invitrogen, A33248) were prepared by centrifuging for 30 s at 16,000 × g. Each sample was transferred to a Phasemaker tube and incubated at RT for 5 min, followed by the addition of 200 µL of chloroform. Tubes were shaken by hand, incubated at RT for 10 min, and centrifuged for 5 min at 16,000 × g at 4 °C. The aqueous phase (clear liquid) was transferred to an RNase free tube, an equal volume of 100% ethanol was added, and the samples were briefly vortexed. A column-based nucleic acid purification kit (Zymo Research, R1016) was used to further process the samples with methods modified from the manufacturer’s instructions. The sample was added 600 µL at a time to the column and centrifuged for 1 min at 12,000 × g, this was repeated until the entire sample was used. All RNA wash/prep buffer steps were performed by adding the buffer to the column and centrifugation for 1 min at 12,000 × g. After the samples were loaded onto the columns, samples were washed with 400 µL of RNA wash buffer. A 1X DNase I cocktail (DNase I from Thermo Scientific FEREN0521; Reaction buffer with MgCl_2_ from Thermo Scientific FERB43) was added to the column, incubated for 15 min at RT. After DNase I cocktail was centrifuged through the column, 400 µL of RNA prep buffer was added and passed through the column. Columns were washed with RNA wash buffer twice, 700 µL then 400 µL respectively, then dried for 2 min at 12,000 × g. DNase/RNase free water (15 µL) was added, columns incubated for 1 min at RT, and RNA was eluted by centrifugation (1 min at 10,000 × g). RNA quality and quantity were assessed with an Agilent 2100 Bioanalyzer using an Agilent RNA 6000 Pico Kit (5067-1513). Before storing RNA at −80 °C, RNA was diluted to 1 ng/µL with DNase/RNase free water and aliquoted.

#### Droplet digital PCR

RNA was thawed, and 2 ng was used with iScript Reverse Transcription Supermix for cDNA synthesis (Bio-Rad, 1708841). cDNA synthesis was performed in a thermocycler with the following settings: 5 min at 25 °C, 20 min at 46 °C, 1 min at 95 °C, hold at 4 °C (constant lid temperature of 105 °C). In case storage at −20 °C was necessary, all cDNA was diluted with 2X cDNA storage buffer (equal parts 10 mM Tris HCl (pH 7.5) and 0.1 mM EDTA pH (8.0)). To prepare samples for droplet digital PCR (ddPCR), cDNA was thawed if needed on ice and master mix containing 2X ddPCR Supermix for Probes (Bio-Rad, 186-3026) and the 20X Taqman primer probe sets was made. Probes used for mice were *Snca* (Applied Biosystems #4331182, Mm01188700_m1, FAM-MGB); and two different reference probes, *Gapdh* (Applied Biosystems #4331182, Mm99999915_g1, VIC-MGB), and *Rpl13* (Applied Biosystems #4331182, Mm02342646_g1, VIC-MGB). Results normalized to *Rpl13* can be found in Supplementary Fig. 8. Probes used for rats were *Snca* (Applied Biosystems #4331182, Rn00569821_m1, FAM-MGB) and the reference probe was *Gapdh* (Applied Biosystems #4331182, Rn01749022_g1, VIC-MGB). For all Taqman primer probe sets used, the probe spanned across an exon-exon junction. Equal parts cDNA and master mix were added to tubes, mixed, briefly centrifuged, and 20 µL added to the sample wells of DG8 droplet generator cartridges (Bio-Rad, 1864008). To the oil wells in the cartridge, 70 µL of droplet generation oil (Bio-Rad, 1863005) was added. A rubber gasket (Bio-Rad, 1863009) was secured over the cartridge, and a QX droplet generator (Bio-Rad, 186-4002) used to produce RNA containing droplets. From the droplet well of the cartridge, 40 µL of droplets are transferred to a 96-well plate (Bio-Rad, 12001925). When all samples are transferred, the plate was sealed with pierceable foil (Bio-Rad, 181-4040) by a plate sealer (Bio-Rad, 181-4000). Plates were transferred to a thermocycler (Bio-Rad, C1000), with the following settings: 10 min at 95 °C, 39 cycles (30 s at 94 °C, 1 min at 60 °C), 10 min at 98 °C, hold at 12 °C (constant lid temperature of 105 °C). After PCR, plates are transferred to the QX200 droplet reader (Bio-Rad, 1864003), and results analyzed with QuantaSoft software. For all samples, the gene of interest was normalized to the reference gene. *Gapdh* was chosen as one of the reference genes as it is a conventionally used housekeeping gene and protein, and shows high and consistent mRNA expression in most tissue. An additional reference gene, *Rpl13a*, was also used because the related gene *Rpl13* was used in Mittal et al.^37^, which originally reported the effects of clenbuterol on *Snca* mRNA and α-syn protein. Use of different reference genes for normalization did not affect the overall results.

#### Sample preparation for western blots and ELISAs

Sample preparation to examine α-syn protein was performed in two steps, an initial weak lysis as performed in Mittal et al.^37^, and a second strong lysis step on the remaining pellet. Frozen tissue punches removed from the −80 °C, 100 µL and 200 µL of weak lysis buffer (320 mM sucrose, 5 mM NaF, 1 mM Na_3_VO_4_, 10 mM Tris (pH 7.4), 1 mM EGTA, 1 mM EDTA) were added to the SN and the striatum respectively. Tissue was homogenized with a disposable pestle, incubated on ice for 10 min and centrifuged for 10 min at 10,000 × g at 4 °C. The supernatant was transferred to a new tube and centrifuged again for 10 min at 10,000 × g at 4 °C, and supernatant collected, with only a small pellet remaining if any. RIPA lysis buffer (Santa Cruz Biotechnology, sc-24948) equal to the original amount of weak lysis buffer was added to the pellet remaining from the initial centrifugation of the weak lysis fraction. The pellet in RIPA buffer was homogenized with 2–4 bursts of a probe sonicator (Qsonica Q125), 2 mm diameter probe (QSonica. 4423), 1 s pulses at an amplitude set at 30%. Samples in RIPA buffer were centrifuged for 10 min at 10,000 × g at 4 °C, and supernatant collected, though little to no pellet was present. A bicinchoninic acid (BCA) assay (Fisher, 23223, bicinchoninic acid; Fisher, 23224, copper sulfate) was used to estimate protein quantity in the weak and strong (RIPA) lysis buffer fractions before storage at −80 °C.

#### Western blots

A 3X sample buffer was made (140 µl 50% Glycerol/0.1 Bromophenol Blue, 40 µl 10% SDS, and 20 µl Beta-mercaptoethanol), and added to 5–10 µg of protein of each sample. Samples were incubated at 100 °C for 5 min, cooled on ice, briefly mixed and centrifuged, and loaded on a 4–20% Criterion TGX 26 well precast gel (Bio-Rad, 5671095) in running buffer (3.03% Trizma base, 14.4% glycine, 1% SDS). A Precision Plus Protein Dual Xtra ladder (Bio-Rad, 161-0377), was used because the range of recombinant protein markers (2–250 kD) included markers around 14 kD, where α-syn is predicted to be present. Gels were run at constant voltage, starting at 90 V for 45 min, then 130 V until the dye front had almost run off the gel. Before the gel was finished running, membranes were prepared for transfer. For α-syn blots, a 0.45 µm nitrocellulose membrane (Bio-Rad, 1620167) and blot paper (Bio-Rad, 1620118) were soaked for at least 5 min in transfer buffer (3.03% Trizma base, 14.4% glycine, 20% methanol). For all other proteins, an Immobilon-FL membrane (Millipore, IPFL00010) was activated for 30 s in 100% methanol, then soaked in transfer buffer instead of the nitrocellulose membrane. A wet transfer was run at constant current, 400 mA, for 45 min. Immediately after the transfer was complete, membranes were removed and only in the case of the α-syn blots, fixed in 0.4% paraformaldehyde in 1X tris-buffered saline (TBS) (pH 7.4) for 30 min. Membranes were washed 2 × 5 min with TBS. Membranes were incubated in Revert Protein Dye (LI-COR 926-11021) for 5 min, washed 2 × 30 s with a solution of 6.7% glacial acetic acid and 30% methanol, rinsed with ddH_2_O, and imaged at 700 nm on a LI-COR Odyssey CLx for total protein. Membranes were removed, washed 2 × 5 min with TBS, and blocked for 1 h in 1:1 TBS-Tween (0.1%):StaringBlock T20 blocking buffer (Thermo Scientific, 37543). Membranes were incubated in primary antibody in 1:1 TBS-Tween (0.1%):StaringBlock T20 blocking buffer overnight at 4 °C. Primary antibodies used were: 1:1,000 rabbit anti-α-syn (Abcam, ab212184, Lot#GR3185934), 1:500 mouse anti-α-syn (Millipore, MABN1817, Lot#3099686), 1:500 rat anti-DAT (Millipore, MAB369, Lot#3258730), 1:1,000 rabbit anti-DAT (Sigma-Aldrich, D6944, Lot#087M4786V), 1:1,000 rabbit anti-β2AR (Invitrogen, MA5-32570, Lot#UH2832117), 1:500 rabbit anti-β2AR phosphorylated at serine 355 (Invitrogen, PA5-38403, Lot#UI2847382), and 1:500 rabbit anti-β2AR phosphorylated at serine 346 (Invitrogen, PA5-36784, Lot#UH2832047). Membranes were washed 3 × 5 min with TBS-Tween (0.1%), and incubated in secondary antibody for 1 h at RT in the dark. Secondary antibodies used were: 1:15,000 donkey anti-rabbit IRDye 800CW (LI-COR, 926-32213, Lot#C60322-03), 1:15,000 goat anti-mouse IRDye 800CW (LI-COR, 926-32210, Lot#C20808-02), or 1:15,000 goat anti-rat IRDye 800CW (LI-COR, 926-32219, Lot#C90813-13). Membranes were kept in the dark and washed 3 × 5 min with TBS-Tween (0.1%), 1 × 5 min in TBS, and imaged on a LI-COR Odyssey CLx for the protein of interest. Band densitometry was performed using LI-COR Image Studio Lite (Version 5.2). Boxes were drawn around the total protein stain and the corresponding target band to obtain the total protein signal and the target band signal in each lane. The signals from each total protein stain lane were divided by the highest total protein stain lane signal to calculate the lane normalization factor for each lane. The normalized signal for each sample was calculated by dividing the target band signal by the corresponding lane normalization factor. Examples of representative full-length lanes from western blot (total protein loading control and target protein) are in Supplementary Figs. 9 and 10.

#### ELISAs

ELISAs were performed using a premade colorimetric mouse α-syn sandwich ELISA kit (LS Bio, LS-F6284-1) following manufacturer’s instructions. Samples were diluted 1:20 for SN, 1:20 for striatum, and 1:25 for plasma in sample diluent. Standards and samples were run in duplicate. A BCA assay was performed using the remaining diluted samples from the SN and striatum, and used to normalize the results to protein. Absorbance at 450 nm was read with a Synergy H1 Hybrid Multi-Mode Reader.

#### RNAScope (in situ hybridization)

Forty-micrometer-thick coronal brain tissue sections were washed in TBS with Triton X-100 (TBS-TX) and incubated in hydrogen peroxide from the RNAscope Pretreatment Kit (Advanced Cell Diagnostics, Hayward, CA; 322330) for 1 h. Sections were washed in TBS and then mounted on VistaVision HistoBond slides (VWR, Randor, PA; 16004-406) and placed on a slide warmer at 60 °C overnight. Slides were then incubated for 10 min in ACD Biosciences target retrieval buffer at 99 °C and then washed twice in water. Tissue was outlined with Pap Pen (Abcam, Cambridge, UK; ab2601), incubated with ACD protease plus in a hybridization oven at 40 °C for 15 min, washed twice in water, and incubated with the probe for rat *Adrb2* (Cat No: 468131, Advanced Cell Diagnostics, Hayward, CA; 457731) for 2 h in the hybridization oven at 40 °C, followed by washes in ACD wash buffer. Six amplification steps with the amplification buffers (Advanced Cell Diagnostics, Hayward, CA; 322300) were then performed in alternating 30- and 15-min incubation intervals in the hybridization oven per manufacturer’s instructions. Tissue was developed using the supplied DAB reagent, washed in TBS-TX, taken through ascending ethanol washes, and cleared with xylenes. Slides were coverslipped with Cytoseal 60 and imaged with a Nikon Eclipse 90i microscope with a QICAM camera (QImaging, Surrey, British Columbia, Canada).

#### Mass spectrometry plasma and CSF

All solvents were of LC-MS grade and purchased from Fisher Scientific. All other chemicals were purchased from Sigma-Aldrich. Spin filters were purchased from Millipore Sigma. 100 μL of rat plasma and 50 μL of mouse plasma was used per sample. Plasma was precipitated with 4:1 ice-cold acetone at −20 °C overnight. After centrifugation (18,000 × *g*, 2 min), supernatant was removed, and dried to completion (30 °C using a vacuum centrifuge). Samples were resuspended in 300 µL of 0.1% formic acid. Insoluble particulates were removed by filtration with a 3 kDa-cutoff spin filter. Flow-through was dried to completion and resuspended in 20 μL 0.1% formic acid, 2% ACN, with 2.5 ng/mL heavy clenbuterol-d9. Calibration curves were generated by mixing untreated plasma from 5 animals (rat or mouse) and spiking the mixed plasma with clenbuterol at concentrations of 0.06, 0.2, 0.6, 2.0, 6.0, 20.0, 60.0, and 200.0 ng/mL. All experiments were conducted on a Thermo Scientific™ Q Exactive™ HF-X Hybrid Quadrupole-Orbitrap™ Mass Spectrometer with a Thermo Scientific™ UltiMate™ 3000 UHPLC system. Each analysis consists of three, 15-min reverse-phase gradients with 2 µl sample injections. Separations use a 15 cm C18 EasySpray column. Samples are loaded onto a 5 mm C18 trap cartridge for online desalting prior to chromatographic separation. Samples were acquired in triplicate.

The mass spectrometry analysis utilized the PRM scan mode for isolating and fragmenting clenbuterol (277.08690 m/z) and heavy clenbuterol (286.14339 m/z). Fragment mass spectra were acquired at 15,000 resolution (200 m/z), with an AGC of 2e5, and a maximum injection time of 100 ms. Peptides were fragmented with stepped collision NCE 25, 50 and an isolation window of 1.3 m/z. Electrospray voltage was 1.9 kV. Peak picking and area calculations were performed by Skyline version 4.2. All peaks are manually validated. The summed peak areas of clenbuterol fragments 203.01373 m/z, 168.044877 m/z, and 132.068199 m/z, normalized to the summed peak areas of heavy clenbuterol fragments 204.020006 m/z, 169.051154 m/z, and 133.074476 m/z are used for quantitation.

#### Mass spectrometry bound and unbound in tissue

All solvents were LC-MS grade and purchased from Fisher Scientific. All other chemicals were purchased from Sigma-Aldrich. Spin filters were purchased from Millipore Sigma. 5–10 mg punches of brain tissue were sequestered into “bound” and “unbound” fractions. The “bound” fractions represent protein-bound clenbuterol, such as the interaction with β2AR. Preparation of “Total”, “Unbound”, and “Bound” fractions for each sample is as follows. All manipulations were performed on ice. Samples were lysed by sonication for 10 s in 200 μL of 25 mM ammonium bicarbonate (AMBIC), pH 8. 10 μL of supernatant was removed as a representation of “Total” clenbuterol and set aside. Samples were centrifuged (18 K × G, 10 min). The remaining supernatant was spin filtered (3 kDa 18 K × G, 10 min) without rinsing. Flow-through was collected as “Unbound” fractions. Material retained on the filter was collected by inverted spin (18 K × G, 2 min) as “Bound” fractions. The pelleted brain material was extracted with 200 μL of 60% ACN, 30% MeOH, 10% 25 mM AMBIC, pH 8, sonicated for 10 s, and centrifuged (18 K × G, 10 min). Supernatant was removed and combined with the “Bound” fractions. Proteins from “Total”, “Unbound”, and “Bound” fractions were precipitated with 5:1 ice-cold acetone at −20 °C overnight. After centrifugation (18 K × G, 10 min), supernatant was removed and dried to completion (30 °C using a vacuum centrifuge). “Unbound” fractions were resuspended in 20 μL 0.1% FA, 2% ACN, with 2.5 ng/mL heavy clenbuterol-d9. “Bound” fractions were resuspended in 20 μL 0.1% FA, 2% ACN, with 2.5 ng/mL heavy clenbuterol-d9. 180 μL 0.1% FA was added, spin filtered, dried to completion, and resuspended in 20 μL 0.1% FA, 2% ACN.

“Total” fractions were resuspended in 200 μL 0.1% formic acid, spin filtered, dried to completion, and resuspended in 20 μL 0.1% FA, 2% ACN, with 2.5 ng/mL heavy clenbuterol-d9. “Unbound” and “Bound” calibration curves were generated by combining 10 mg untreated brain tissue from 10 mice, sonicating in 2 mL of buffer, aliquoting lysate into ten samples, and removing 10 μL from each aliquot. Aliquots were separated into “Unbound” and “Bound” fractions by spin filtration as described above. Each fraction was then spiked with clenbuterol at concentrations of 0.3, 1.0, 3.0, 10.0, 30.0, 100.0, 300.0 pg, 1.0, 3.0, and 10.0 ng. Clenbuterol was extracted as described above.

The “Total” calibration curve was generated by combining 10 mg untreated brain tissue from eight mice, sonicating in 1.6 mL of buffer, aliquoting lysate into 8 samples, and spiking with clenbuterol at concentrations of 3.0, 10.0, 30.0, 100.0, 300.0 pg, 1.0, 3.0, and 10.0 ng.

#### Statistics

Statistical analysis was performed using GraphPad Prism version 7.03, and significance for all cases was performed using *α* ≤ 0.05. Outliers were assessed using the absolute deviation from the median method^78^, with a “very conservative” difference of 2.5X median absolute deviation used as the exclusion criteria. Experiments with two groups (i.e. saline control and clenbuterol) were analyzed using a two-tailed *t*-test. Experiments with multiple groups at a single timepoint (i.e. saline control, 10, 20, and 40 mg/kg clenbuterol) were analyzed with a one-way ANOVA and post-hoc Tukey tests for multiple comparisons. Experiments with multiple groups at the 1-day and 1-week timepoint were analyzed with a two-way ANOVA and post-hoc Tukey tests for multiple comparisons. Analysis of weight changes was performed with a two-way repeated measures ANOVA and post-hoc Sidak test (mice) or Tukey test (rat) for multiple comparisons. All group means, standard errors, and statistical test information for all of the results can be found in the supplementary statistics document.

### Biogen lab methods

#### Cell culture

Sprague-Dawley E18 primary rat cortical neurons were plated 50 K cells per well in a 96-well poly-D-lysine coated plate. At DIV11, DMSO vehicle or clenbuterol (1, 5, 10, or 20 µM final well concentration) were added. Two days later, at DIV 13, cells were washed with PBS, then 175 µL of RLT Plus buffer with 0.1% β-mercaptoethanol was added to each well. After combining 2 wells per sample, a Qiagen RNeasy Plus Mini Kit paired with a QiaCube was to extract RNA according to the manufacturer’s instructions. For cDNA synthesis, 16 µL of RNA was added to 4 µL of 5x iScript RT Supermix (Bio-Rad) and placed in a thermocycler (5 min at 25 °C, 30 min at 42 °C, 1 min at 85 °C, hold at 4 °C). Multiplex TaqMan RT-qPCR was used to quantify relative gene expression in a 10 uL reaction volume with a ROX reference dye. A custom rat *Snca* (forward: CTGTACCTGCCCCTCAGCAT; reverse: AGCCTGCTACCATGTATTCACTGTAG; probe: CGGTGCTCCCCTCT) and commercial *Xpnpep1* (Applied Biosystems Rn00590960_m1) primers/probe sets were used. Amplification of *Snca* was normalized to that of *Xpnpep1*. Fold-change values were calculated using the ΔΔCt method and the ratio of drug- to control-treated samples expressed. Data were analyzed with the Thermo Fisher Connect cloud web tool and Microsoft Excel. CellTiter-Glo Luminescent Cell Viability Assay (Promega) was used according to the manufacturer’s instructions to assess toxicity of treatments.

#### Mouse study

C57BL/6 J mice (*n* = 24: ten saline and 14 clenbuterol) received a single *i.p*. injection of 10 mg/kg clenbuterol or equal volume of saline control. Animals were euthanized 24 h after dosing, the brain removed, and flash frozen in liquid nitrogen. Brains were sectioned on a cryostat and frozen punches of the SN collected. Tissue punches were homogenized in QIAzol using an Omni Bead Disruptor (45 s on, 30 s off, 45 s on). After 5 min at room temperature, 200 uL of chloroform was added and samples were vortexed for 15 s. After 3 min at room temperature, samples were centrifuged at 12,000 rpm for 15 min at 4 °C. 400 uL of the resulting aqueous layer was combined with an equal volume of 70% ethanol and RNA was subsequently extracted using the RNeasy Mini Kit (Qiagen) according to the manufacturer’s instructions. Reverse transcription of 300 ng RNA was performed in a 20 uL reaction volume using SuperScript VILO cDNA Synthesis Kit (Thermo Fisher). This reaction volume was diluted 1:2 with water prior to loading 2uL into a 10 uL qPCR reaction containing 5 uL TaqMan Gene Expression Master Mix (Thermo Fisher, 4369016) and 0.2 uM gene-specific TaqMan assay. TaqMan primers/probe, identical to those used in Mittal et al^37^, were used for *Snca* (Applied Biosystems Mm01188700_m1), which was normalized to either *Rpl13a* (Applied Biosystems Mm02342645_g1), *Actb* (Applied Biosystems Mm00607939_s1), or *Ubc* (Applied Biosystems Mm01198158_m1). For all samples, the gene of interest was normalized to the reference gene or the geometric mean of the three reference genes. *ActB* and *Ubc*, were selected based on their stable expression, known use as housekeeping genes, and because of their prior use in the original report on which this study is based^37^. Similarly, *Rpl13a*, was also used because the related gene *Rpl13* was used as one of the reference genes in the previous report^37^. Use of different reference genes for normalization did not affect the overall results. Results using housekeeping genes not shown in the main manuscript can be found in Supplementary Fig. 8.

### Reporting summary

Further information on research design is available in the Nature Research Reporting Summary linked to this article.

## Supplementary information

Supplementary Figures

Supplemental Statistics File

Reporting Summary Checklist

## Data Availability

Statistical test information for all of the results can be found in the supplementary statistics document. The data that support the findings in this article are available on reasonable request from the corresponding author.
