# Supplementary material for: Beta2-adrenoreceptor agonist clenbuterol produces transient decreases in alpha-synuclein mRNA but no long-term reduction in protein
Source: NPJ Parkinsons Dis. 2022 May 24;8:61. doi: 10.1038/s41531-022-00322-x (PMC9130326; doi:10.1038/s41531-022-00322-x)
Supplement: Supplementary file 1 — Supplementary Figures [file 41531_2022_322_MOESM1_ESM.pdf]

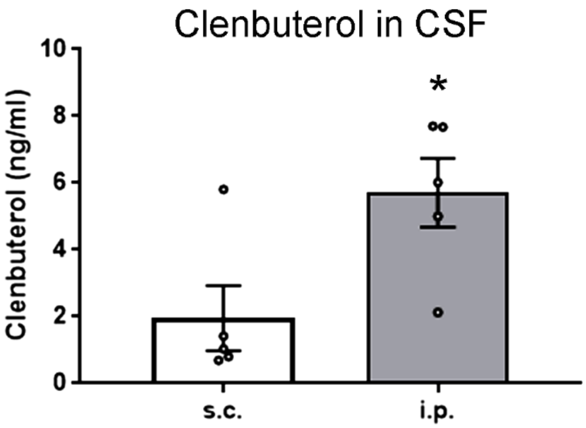

Supplementary Figure 1. Intraperitoneal route of administration yields higher clenbuterol levels in the CSF. Rats received 10 mg/kg clenbuterol or vehicle either by subcutaneous (s.c.) or intraperitoneal (i.p.) injection. CSF was collected 24 h after clenbuterol administration. Columns indicate the group means, circles represent individual data points (n=5 per group), error bars represent ± 1 standard error of the mean. An asterisk represents significance ($p \leq 0.05$).

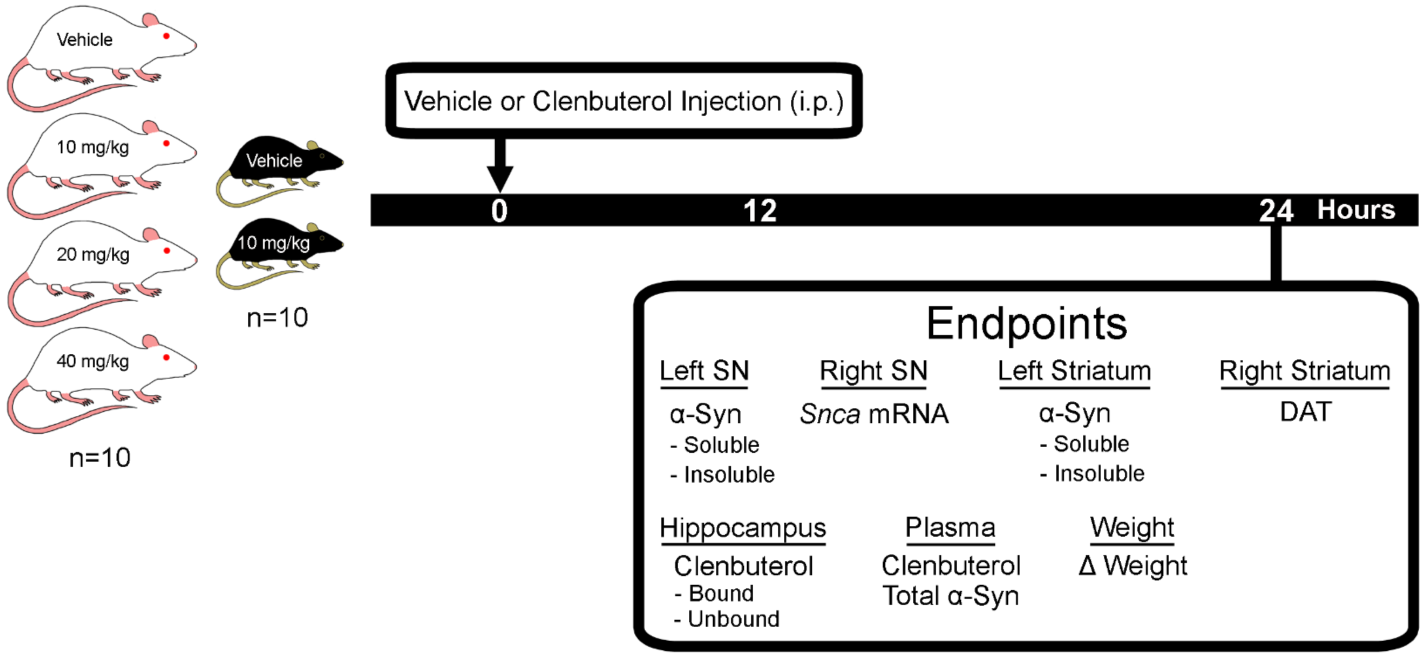

Supplementary Figure 2. Diagram of the one day single clenbuterol administration paradigm. Rats (white) received 10, 20, or 40 mg/kg clenbuterol or vehicle; and mice (black) received 10 mg/kg clenbuterol or vehicle via a single intraperitoneal (i.p.) injection on day 0. At 24 h post-injection, animals were given a pentobarbital overdose, cardiac blood collected, and blood flushed from the brains via perfusion with heparinized 0.9% saline. Blood was centrifuged to collect plasma. Brains were flash-frozen in 2-methyl butane and stored at -80°C. Frozen brains were dissected on a cryostat to collect the hippocampus, and the SN and striatum from each hemisphere, Endpoints measured are listed above for each brain region.

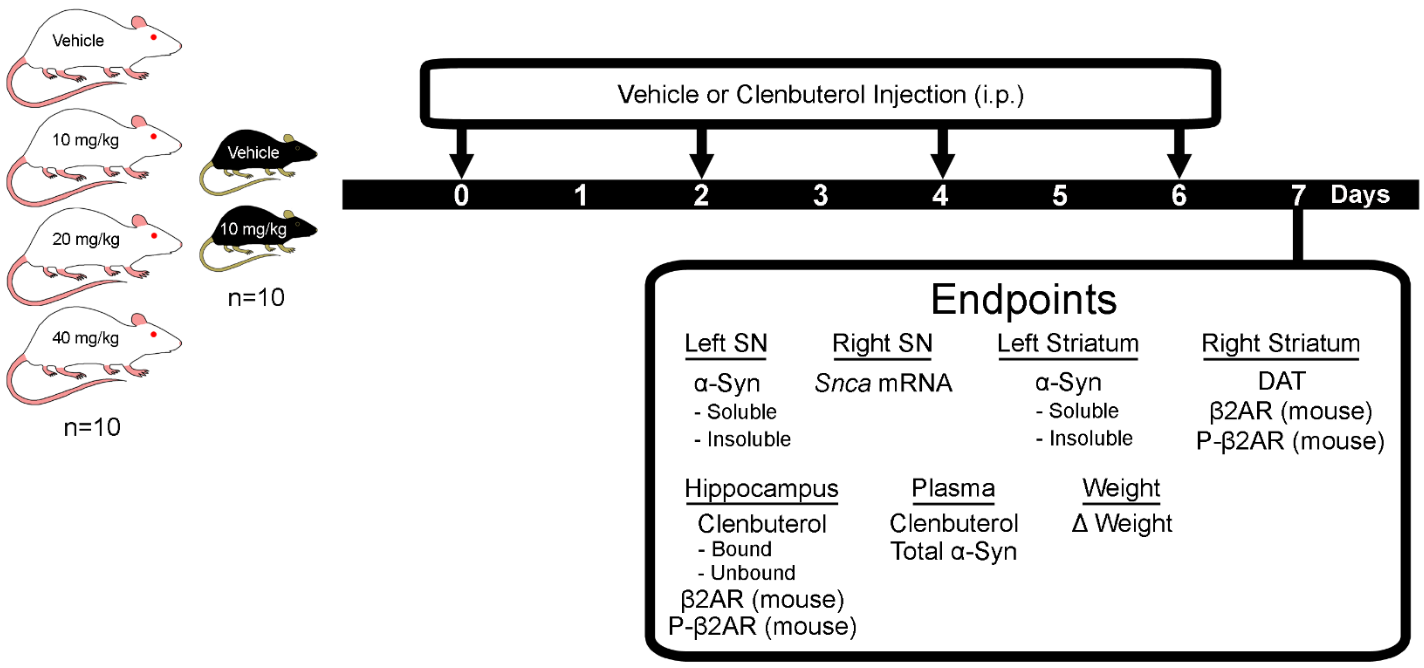

Supplementary Figure 3. Diagram of the one week multiple clenbuterol administration paradigm. Rats (white) received 10, 20, or 40 mg/kg clenbuterol or vehicle; and mice (black) received 10 mg/kg clenbuterol or vehicle via an intraperitoneal (i.p.) injections every 48 h starting on day 0. Animals were weighed prior to each injection and before they were sacrificed to calculate the change in weight (Δ weight). At 24 h after the final injection on day 6, animals were given a pentobarbital overdose, cardiac blood collected, and blood flushed from the brains via perfusion with heparinized 0.9% saline. Blood was centrifuged to collect plasma. Brains were flash-frozen in 2-methyl butane and stored at -80°C. Frozen brains were dissected on a cryostat to collect the hippocampus, and the SN and striatum from each hemisphere, Endpoints measured are listed above for each brain region.

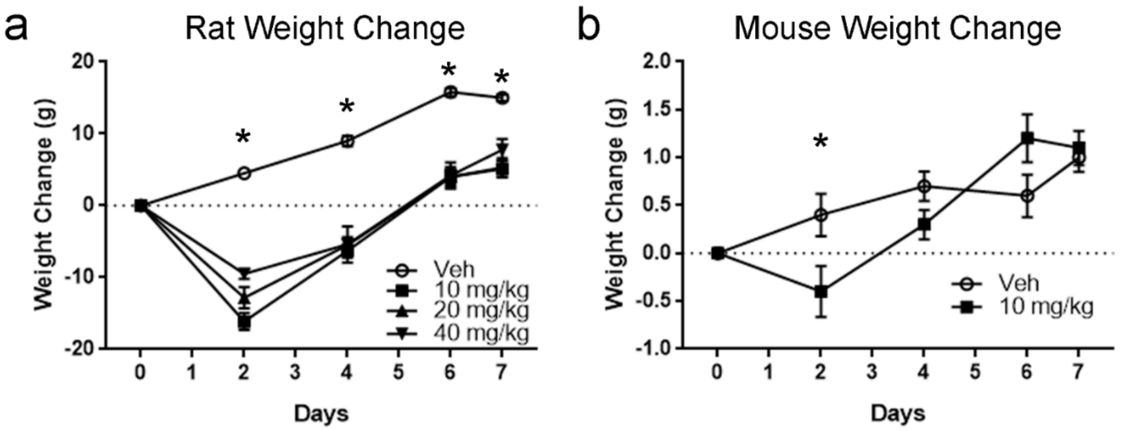

Supplementary Figure 4. Weight change associated with clenbuterol in rats and mice. Rats (10, 20, or 40 mg/kg) and mice (10 mg/kg) received clenbuterol or equal volume of saline every other day. Prior to each injection and end-point euthanasia, animals were weighed. The change from the initial weight was calculated for each animal, then averaged by group for each weigh time. Average change from initial weight from **a.** rats and **b.** mice were plotted over time. Symbols represent the group means (n=10 per group) and error bars represent ± 1 standard error of the mean. In some cases, the error is too small to be represented by error bars on the graphs. An asterisk represents significance ($p \leq 0.05$).

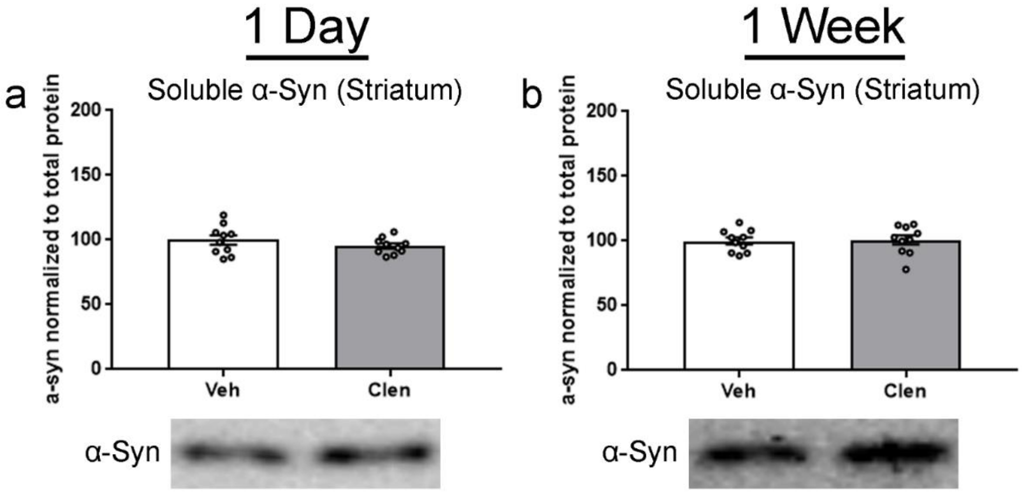

Supplementary Figure 5. α-syn detected using Millipore MABN1817 primary antibody. Mice received 10 mg/kg clenbuterol (clen) or saline vehicle (veh) following the **a.** one day or **b.** one week dosing paradigm. Soluble α-syn from the mouse striatum was measured by western blot and graphed as the percent of control. Representative western blots for α-syn are shown below each graph respectively. Columns indicate the group means, circles represent individual data points (n=10 per group), error bars represent ± 1 standard error of the mean.

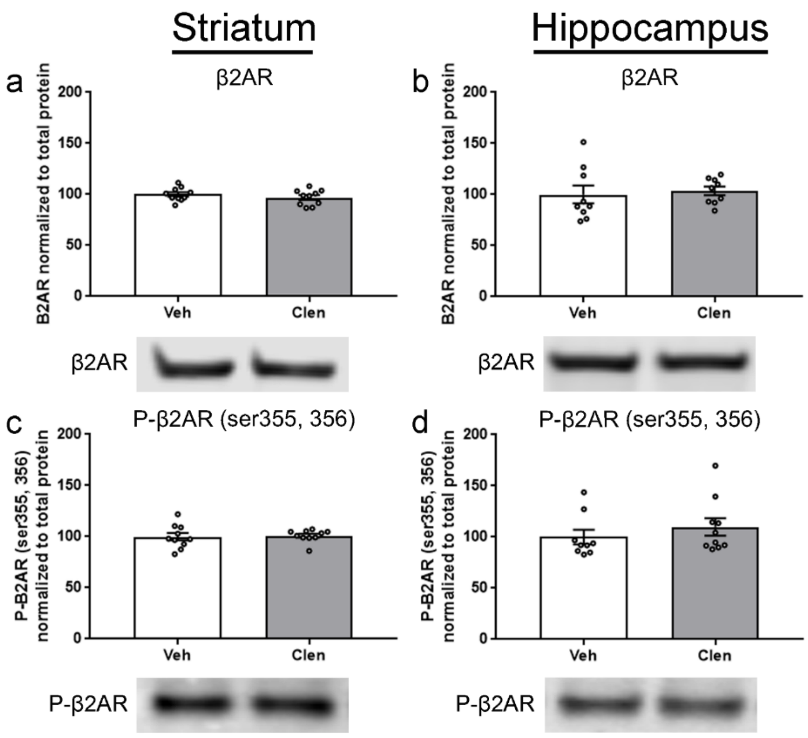

Supplementary Figure 6. Examination of phosphorylated and total β2AR in the striatum and hippocampus. Mice received intraperitoneal injections of 10 mg/kg clenbuterol (clen) or saline vehicle (veh) every 48 h. Tissue was collected on the seventh day, 24 h after the final injection and homogenized in a strong lysis buffer (RIPA). Total β2AR protein in the **a.** striatum and **b.** hippocampus. β2AR phosphorylated at serine 355 and 356 in the **c.** striatum and **d.** hippocampus. All protein was measured via western blot, graphed as percent of control, and representative blots are shown below the respective graph. Columns indicate the group means, circles represent individual data points (n=10 per group before outlier removal), error bars represent ± 1 standard error of the mean. An asterisk represents significance ($p \leq 0.05$). Outliers were removed based on the absolute deviation from the median method. In **b**, one sample was removed from the veh and clen groups. In **d**, one sample was removed from the veh group. No other outliers were removed.

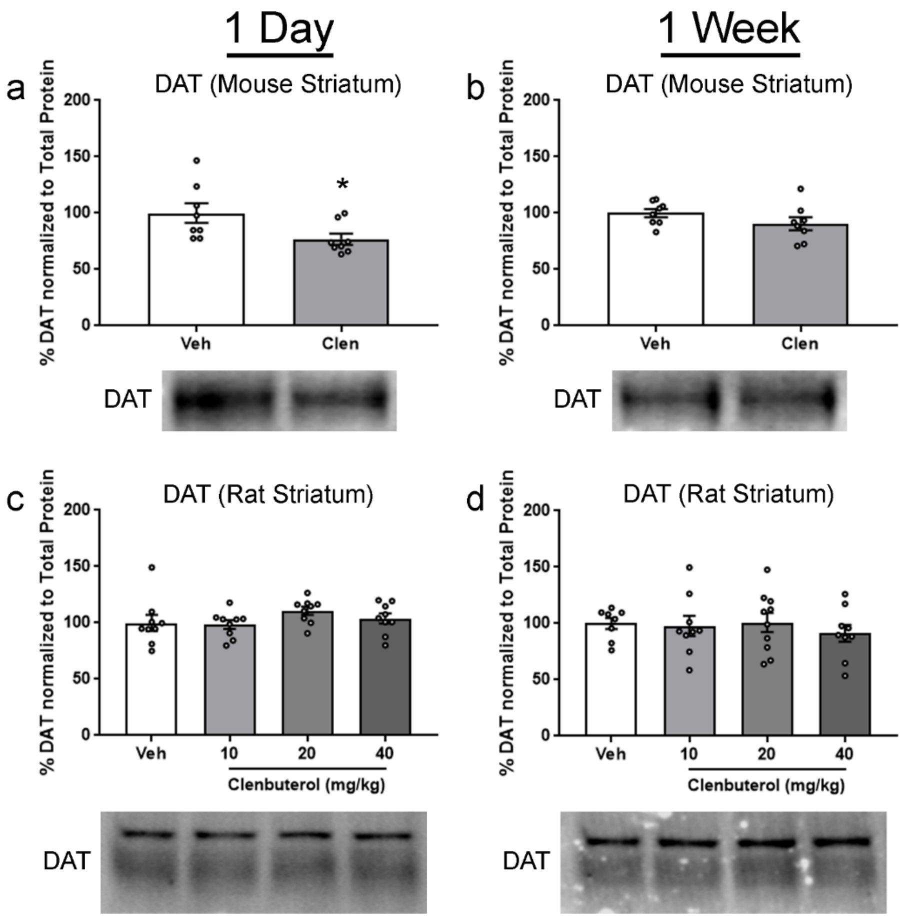

Supplementary Figure 7. Evaluation of DAT in clenbuterol treated mice and rats. Mice received 10 mg/kg clenbuterol (clen) or saline vehicle (veh), and rats received 10, 20, or 40 mg/kg clenbuterol (clen) or saline vehicle (veh) following the one day or one week dosing paradigm. Dissected tissue was homogenized in a strong lysis (RIPA) buffer to isolate total DAT from the striatum. DAT at **a.** one day and **b.** one week isolated from the mouse striatum. DAT at **c.** one day and **d.** one week isolated from the rat striatum. All protein was measured by western blot and graphed as a percent of control. Representative western blots for DAT are shown below each graph respectively. Columns indicate the group means, circles represent individual data points (n=10 per group before outlier removal), error bars represent ± 1 standard error of the mean. Outliers were removed based on the absolute deviation from the median method. In **a** and **b**, two samples were removed from the veh and clen groups. In **c**, one sample was removed from each group. In **d**, two samples were removed from the veh group, and one sample removed from the 10 and 40 mg/kg groups. No other outliers were removed.

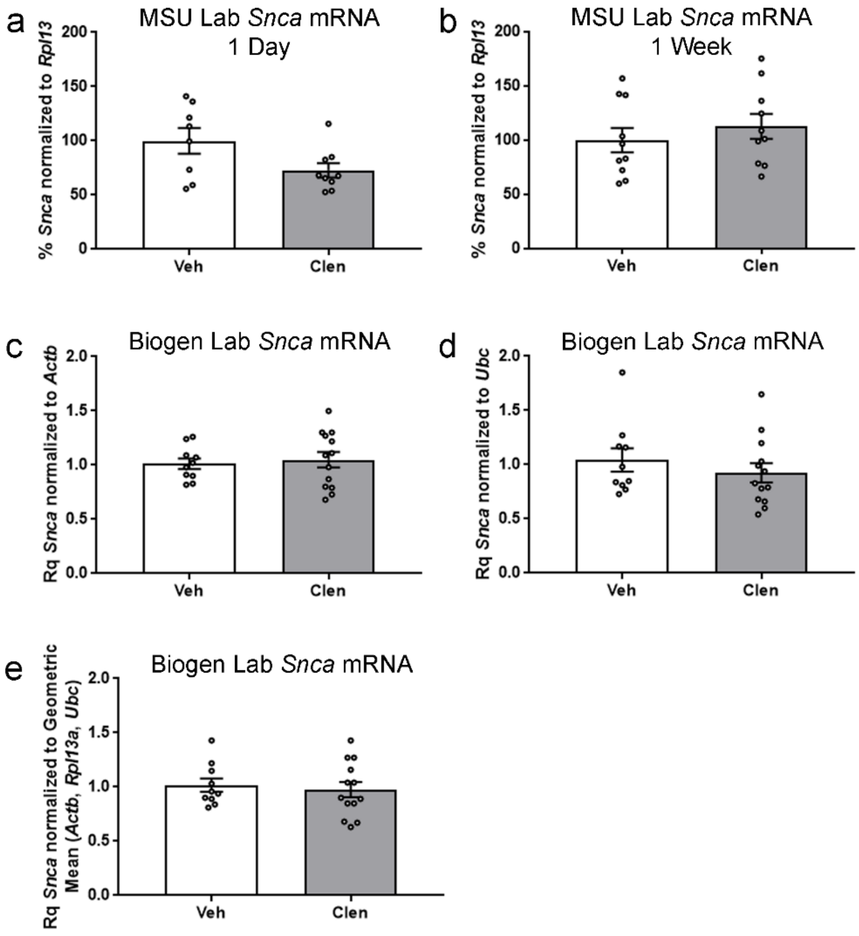

Supplementary Figure 8. *Snca* transcript from mice normalized to different housekeeping genes. In addition to the normalization of *Snca* to *Gapdh* (MSU Lab) and *Rpl13a* (Biogen Lab), other housekeeping genes were used. In the MSU lab, *Snca* measured by ddPCR was also normalized to *Rpl13* in the **a.** one day and **b.** one week paradigms. In the Biogen lab, *Snca* measured by RT-qPCR in the one day paradigm was also normalized to **c.** *Actb*, **d.** *Ubc*, and **e.** the geometric mean of the mRNA of *Actb*, *Rpl13a*, and *Ubc* housekeeping genes. End results of the degree of change or lack thereof did not change with the use of different housekeeping genes. Columns indicate the group means, circles represent individual data points, error bars represent ± 1 standard error of the mean. Outliers were removed based on the absolute deviation from the median method. In **a**, two samples were removed from the veh, and one sample removed from the clen groups. No other outliers were removed.

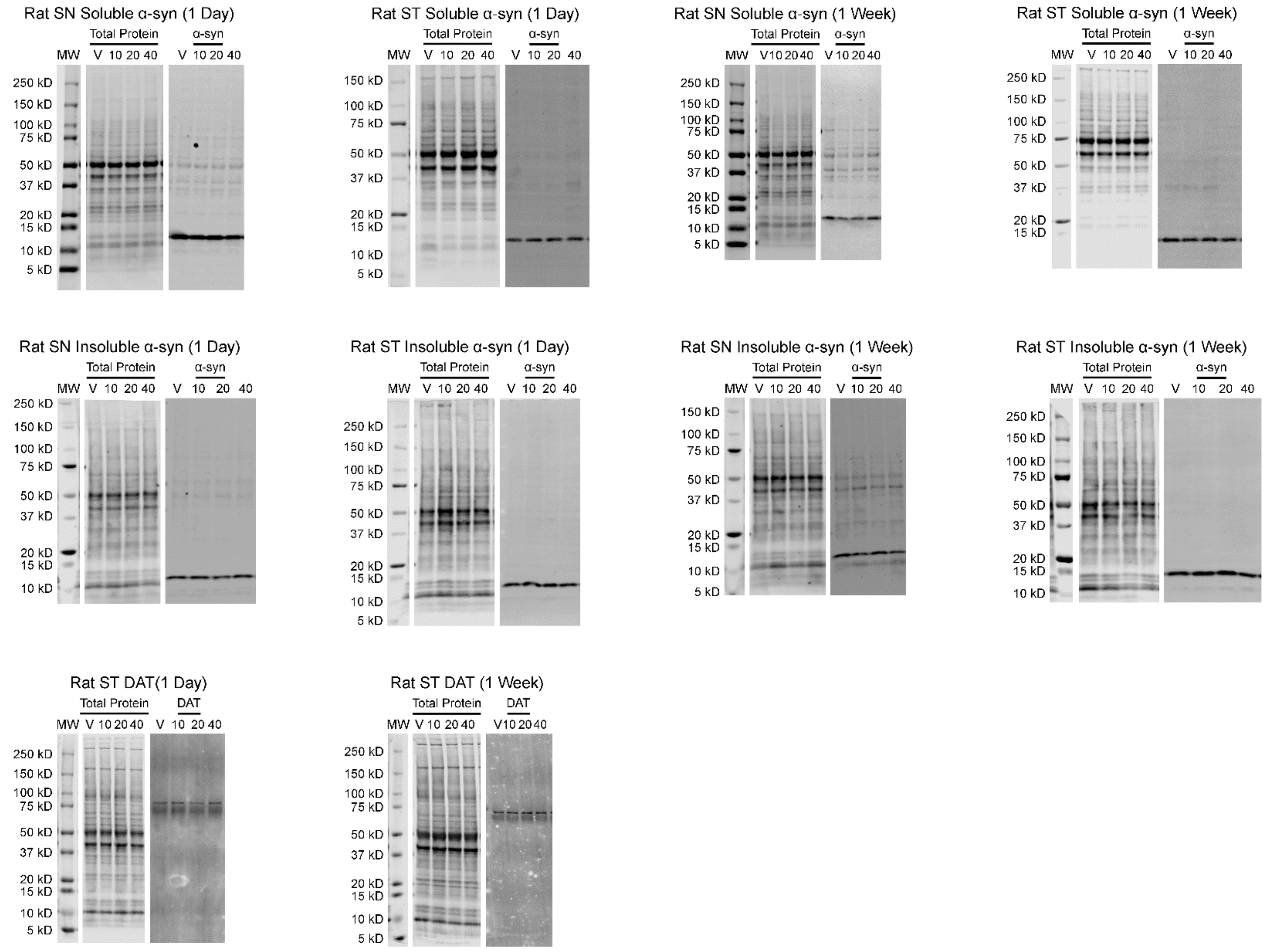

Supplementary Figure 9. Representative western blots images from rat studies. Example images from each rat western blot endpoint are displayed. Shown are both the readily soluble and initially insoluble α-syn fractions from the substantia nigra (SN) and striatum (ST) after 1 day or 1 week of clenbuterol (clen) administration. Also shown are blots of the dopamine transporter (DAT) from the ST after 1 day or 1 week of clenbuterol (clen) treatment. Within each set, the protein ladder denoting molecular weights (MW) in kD, the total protein stain loading control, and the target protein of interest are shown. Treatment in each lane is denoted at the top of each image, showing vehicle (V), or treatment with 10 mg/kg (10), 20 mg/kg (20), or 40 mg/kg (40) of clenbuterol (clen). Target protein (band) size can be estimated based on the protein ladder.

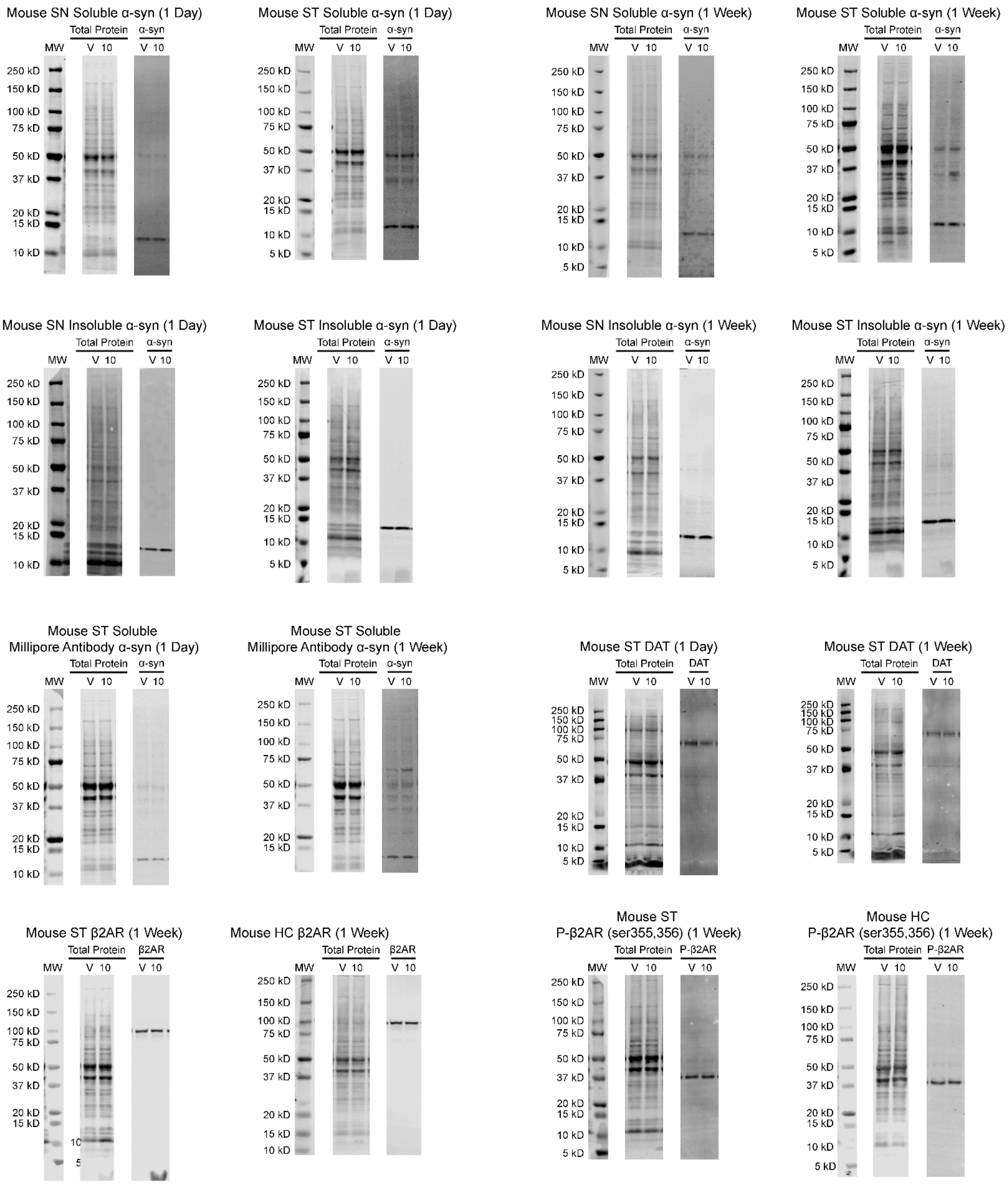
Mouse SN Soluble α-syn (1 Day)
Total Protein
α-syn
MW
V 10
V 10
250 kD
150 kD
100 kD
75 kD
50 kD
37 kD
20 kD
15 kD
10 kD
Mouse ST Soluble α-syn (1 Day)
Total Protein
α-syn
MW
V 10
V 10
250 kD
150 kD
100 kD
75 kD
50 kD
37 kD
20 kD
15 kD
10 kD
5 kD
Mouse SN Soluble α-syn (1 Week)
Total Protein
α-syn
MW
V 10
V 10
250 kD
150 kD
100 kD
75 kD
50 kD
37 kD
20 kD
15 kD
10 kD
5 kD
Mouse ST Soluble α-syn (1 Week)
Total Protein
α-syn
MW
V 10
V 10
250 kD
150 kD
100 kD
75 kD
50 kD
37 kD
20 kD
15 kD
10 kD
5 kD
Mouse SN Insoluble α-syn (1 Day)
Total Protein
α-syn
MW
V 10
V 10
250 kD
150 kD
100 kD
75 kD
50 kD
37 kD
20 kD
15 kD
10 kD
Mouse ST Insoluble α-syn (1 Day)
Total Protein
α-syn
MW
V 10
V 10
250 kD
150 kD
100 kD
75 kD
50 kD
37 kD
20 kD
15 kD
10 kD
5 kD
Mouse SN Insoluble α-syn (1 Week)
Total Protein
α-syn
MW
V 10
V 10
250 kD
150 kD
100 kD
75 kD
50 kD
37 kD
20 kD
15 kD
10 kD
5 kD
Mouse ST Insoluble α-syn (1 Week)
Total Protein
α-syn
MW
V 10
V 10
250 kD
150 kD
100 kD
75 kD
50 kD
37 kD
20 kD
15 kD
10 kD
5 kD
Mouse ST Soluble
Millipore Antibody α-syn (1 Day)
Total Protein
α-syn
MW
V 10
V 10
250 kD
150 kD
100 kD
75 kD
50 kD
37 kD
20 kD
15 kD
10 kD
Mouse ST Soluble
Millipore Antibody α-syn (1 Week)
Total Protein
α-syn
MW
V 10
V 10
250 kD
150 kD
100 kD
75 kD
50 kD
37 kD
20 kD
15 kD
Mouse ST DAT (1 Day)
Total Protein
DAT
MW
V 10
V 10
250 kD
150 kD
100 kD
75 kD
50 kD
37 kD
20 kD
15 kD
10 kD
5 kD
Mouse ST DAT (1 Week)
Total Protein
DAT
MW
V 10
V 10
250 kD
150 kD
100 kD
75 kD
50 kD
37 kD
20 kD
15 kD
10 kD
5 kD
Mouse ST β2AR (1 Week)
Total Protein
β2AR
MW
V 10
V 10
250 kD
150 kD
100 kD
75 kD
50 kD
37 kD
20 kD
15 kD
10
5
Mouse HC β2AR (1 Week)
Total Protein
β2AR
MW
V 10
V 10
250 kD
150 kD
100 kD
75 kD
50 kD
37 kD
20 kD
15 kD
10 kD
Mouse ST
P-β2AR (ser355,356) (1 Week)
Total Protein
P-β2AR
MW
V 10
V 10
250 kD
150 kD
100 kD
75 kD
50 kD
37 kD
20 kD
15 kD
10 kD
5 kD
Mouse HC
P-β2AR (ser355,356) (1 Week)
Total Protein
P-β2AR
MW
V 10
V 10
250 kD
150 kD
100 kD
75 kD
50 kD
37 kD
20 kD
15 kD
10 kD
5 kD

Supplementary Figure 10. Representative western blots images from mouse studies. Example images from each mouse western blot endpoint are displayed. Shown are both the readily soluble and initially insoluble α-syn fractions from the substantia nigra (SN) and striatum (ST) after 1 day or 1 week of clenbuterol (clen) administration. Also shown is soluble α-syn in the ST detected with the Millipore MABN1817 primary antibody, blots of the dopamine transporter (DAT) from ST, and blots for total β2AR and β2AR phosphorylated at serine 355 and 355 from the ST and hippocampus (HC). Within each set, the protein ladder denoting molecular weights (MW) in kD, the total protein stain loading control, and the target protein of interest are shown. Treatment in each lane is denoted at the top of each image, showing vehicle (V), or treatment with 10 mg/kg (10) of clenbuterol (clen). Target protein (band) size can be estimated based on the protein ladder.
