# Supplementary material for: Beta2-adrenoreceptor agonist clenbuterol produces transient decreases in alpha-synuclein mRNA but no long-term reduction in protein
Source: NPJ Parkinsons Dis. 2022 May 24;8:61. doi: 10.1038/s41531-022-00322-x (PMC9130326; doi:10.1038/s41531-022-00322-x)
Supplement: Supplementary file 3 — Reporting Summary Checklist [file 41531_2022_322_MOESM3_ESM.pdf]

nature portfolio

Corresponding author(s): Joseph R. Patterson

Last updated by author(s): Mar 16, 2022

Reporting Summary

Nature Portfolio wishes to improve the reproducibility of the work that we publish. This form provides structure for consistency and transparency in reporting. For further information on Nature Portfolio policies, see our Editorial Policies and the Editorial Policy Checklist.

Statistics

For all statistical analyses, confirm that the following items are present in the figure legend, table legend, main text, or Methods section.

n/a	Confirmed	
☐	☒	The exact sample size (*n*) for each experimental group/condition, given as a discrete number and unit of measurement
☐	☒	A statement on whether measurements were taken from distinct samples or whether the same sample was measured repeatedly
☐	☒	The statistical test(s) used AND whether they are one- or two-sided<br>*Only common tests should be described solely by name; describe more complex techniques in the Methods section.*
☒	☐	A description of all covariates tested
☐	☒	A description of any assumptions or corrections, such as tests of normality and adjustment for multiple comparisons
☐	☒	A full description of the statistical parameters including central tendency (e.g. means) or other basic estimates (e.g. regression coefficient) AND variation (e.g. standard deviation) or associated estimates of uncertainty (e.g. confidence intervals)
☐	☒	For null hypothesis testing, the test statistic (e.g. *F*, *t*, *r*) with confidence intervals, effect sizes, degrees of freedom and *P* value noted<br>*Give P values as exact values whenever suitable.*
☒	☐	For Bayesian analysis, information on the choice of priors and Markov chain Monte Carlo settings
☒	☐	For hierarchical and complex designs, identification of the appropriate level for tests and full reporting of outcomes
☒	☐	Estimates of effect sizes (e.g. Cohen's *d*, Pearson's *r*), indicating how they were calculated

Our web collection on statistics for biologists contains articles on many of the points above.

Software and code

Policy information about availability of computer code

Data collection	NA
Data analysis	NA

For manuscripts utilizing custom algorithms or software that are central to the research but not yet described in published literature, software must be made available to editors and reviewers. We strongly encourage code deposition in a community repository (e.g. GitHub). See the Nature Portfolio guidelines for submitting code & software for further information.

Data

Policy information about availability of data

All manuscripts must include a data availability statement. This statement should provide the following information, where applicable:

- Accession codes, unique identifiers, or web links for publicly available datasets
- A description of any restrictions on data availability
- For clinical datasets or third party data, please ensure that the statement adheres to our policy

Statistical test information for all of the results can be found in the supplemental statistics document. The data that support the findings in this article are available on reasonable request from the corresponding author.

Field-specific reporting

Please select the one below that is the best fit for your research. If you are not sure, read the appropriate sections before making your selection.

☒ Life sciences ☐ Behavioural & social sciences ☐ Ecological, evolutionary & environmental sciences

For a reference copy of the document with all sections, see nature.com/documents/nr-reporting-summary-flat.pdf

Life sciences study design

All studies must disclose on these points even when the disclosure is negative.

Sample size	Power calculations were performed in SigmaPlot 12.0, where significance is $\alpha \leq 0.05$ and power is $\beta \geq 0.80$. Sample sizes selected allow for the exclusion of two rats per group (missed injection, death, etc.) while still maintaining a power of $\beta \geq 0.80$. For all experiments listed, the estimated standard deviation is 15%, based on previous Western blot and stereology data from our lab, as well as qPCR and ChIP-qPCR estimates from the relevant α-syn/clenbuterol literature.
Data exclusions	Outliers were assessed using the absolute deviation from the median method (Leys et al., 2013), with a “very conservative” difference of 2.5X median absolute deviation used as the exclusion criteria.
Replication	Experiments were performed with the appropriate sample sizes based on power calculations. Samples were assessed for similar endpoints which yielded the same results (example: we measured both protein and mRNA for alpha-synuclein). In addition, some sample sets were re-examined using different methods and we saw the same results (example: we measured alpha-synuclein protein by western blot with two different antibodies, and with a commercially available ELISA).
Randomization	Animals were randomly assigned treatment groups by cage at the beginning of the experiments.
Blinding	Animals were randomly assigned to treatment groups and assigned a number. Identities of groups were not reveled to those performing the assessments.

Reporting for specific materials, systems and methods

We require information from authors about some types of materials, experimental systems and methods used in many studies. Here, indicate whether each material, system or method listed is relevant to your study. If you are not sure if a list item applies to your research, read the appropriate section before selecting a response.

Materials & experimental systems

n/a	Involved in the study	
☐	☒	Antibodies
☒	☐	Eukaryotic cell lines
☒	☐	Palaeontology and archaeology
☐	☒	Animals and other organisms
☒	☐	Human research participants
☒	☐	Clinical data
☒	☐	Dual use research of concern

Methods

n/a	Involved in the study	
☒	☐	ChIP-seq
☒	☐	Flow cytometry
☒	☐	MRI-based neuroimaging

Antibodies

Antibodies used	Rabbit anti-α-syn (Abcam, ab212184) Lot: GR3185934<br>Mouse anti-α-syn (Millipore, MABN1817, Clone 2F12) Lot: 3099686<br>Rat anti-DAT (Millipore, MAB369, Clone DAT-Nt) Lot: 3258730<br>Rabbit anti-DAT (Sigma-Aldrich, D6944) Lot: 087M4786V<br>Rabbit anti-β2AR (Invitrogen, MA5-32570) Lot: UH2832117<br>Rabbit anti-β2AR phosphorylated at serine 355 (Invitrogen, PA5-38403) Lot: UI2847382<br>Rabbit anti-β2AR phosphorylated at serine 346 (Invitrogen, PA5-36784) Lot: UH2832047<br>Donkey anti-rabbit IRDye 800CW (LI-COR, 926-32213) Lot: C60322-03<br>Goat anti-mouse IRDye 800CW (LI-COR, 926-32210) Lot: C20808-02<br>Goat anti-rat IRDye 800CW (LI-COR, 926-32219) Lot: C90813-13
Validation	Rabbit anti-α-syn (Abcam, ab212184) product site states it was knockout validated per the product website and validated in lab to be specific to the expected size via western blot.

Mouse anti-α-syn (Millipore, MABN1817) product site states "Clone 2F12 reacted with both monomeric and aggregated forms of alpha-synuclein of human, mouse, and rat species. Clone 2F12 detected both wild-type alpha-synuclein and fPD mutants (Dettmer, U., et al. (2015). Nat. Commun. 6:7314; Dettmer, U., et al. (2013). J. Biol. Chem. 288(9):6371-6385)." Also validated in our lab to validated in lab to be specific to the expected size via western blot.

Rat anti-DAT (Millipore, MAB369), product site states "Recognizes Dopamine transporter, N-terminus. Shows no cross reactivity to the closely related serotonin and norepinephrine transporters (Miller, 1997). Immunolocalization of DAT on paraformaldehyde fixed frozen sections of human brain using MAB369 shows dense punctate staining throughout the caudate, putamen and accumbens (Miller, 1997)." Also validated in lab to be specific to the expected size via western blot.

Rabbit anti-DAT (Sigma-Aldrich, D6944) Product validation information can be found at https://www.sigmaaldrich.com/US/en/product/sigma/d6944. Also validated in lab to be specific to the expected size via western blot.

Rabbit anti-β2AR (Invitrogen, MA5-32570) Product site states "Recombinant rabbit monoclonal antibodies are produced using in vitro expression systems. The expression systems are developed by cloning in the specific antibody DNA sequences from immunoreactive rabbits. Then, individual clones are screened to select the best candidates for production." Also validated in lab to be specific to the expected size via western blot.

Rabbit anti-β2AR phosphorylated at serine 355 (Invitrogen, PA5-38403) Product validation information can be found at https://www.thermofisher.com/antibody/product/Phospho-beta-2-Adrenergic-Receptor-Ser355-Ser356-Antibody-Polyclonal/PA5-38403. Also validated in lab to be specific to the expected size via western blot.

Rabbit anti-β2AR phosphorylated at serine 346 (Invitrogen, PA5-36784) Product validation information can be found at https://www.thermofisher.com/antibody/product/Phospho-beta-2-Adrenergic-Receptor-Ser346-Antibody-Polyclonal/PA5-36784. Also validated in lab to be specific to the expected size via western blot.

Animals and other organisms

Policy information about studies involving animals; ARRIVE guidelines recommended for reporting animal research

Laboratory animals	Three-month-old, male Fischer 344 rats (n=90) were purchased from Charles River Laboratories.<br><br>Eight-week-old male C57BL/6J mice (n=40) were purchased from the Jackson Laboratory.
Wild animals	NA
Field-collected samples	NA
Ethics oversight	All procedures were approved and conducted in accordance with the Michigan State University Institute for Animal Care and Use Committee (IACUC) at Michigan State University

Note that full information on the approval of the study protocol must also be provided in the manuscript.
